# Mutant p53s and chromosome 19 microRNA cluster overexpression regulate cancer testis antigen expression and cellular transformation in hepatocellular carcinoma

**DOI:** 10.1038/s41598-021-91924-7

**Published:** 2021-06-16

**Authors:** Goodwin G. Jinesh, Marco Napoli, Marian T. Smallin, Andrew Davis, Hayley D. Ackerman, Payal Raulji, Nicole Montey, Elsa R. Flores, Andrew S. Brohl

**Affiliations:** 1 Department of Molecular Oncology, H. Lee Moffitt Cancer Center & Research Institute, 12902 USF Magnolia Drive, Tampa, FL 33612 USA; 2 Sarcoma Department, H. Lee Moffitt Cancer Center & Research Institute, 12902 USF Magnolia Drive, Tampa, FL 33612 USA; 3 Cancer Biology and Evolution Program, H. Lee Moffitt Cancer Center & Research Institute, 12902 USF Magnolia Drive, Tampa, FL 33612 USA; 4 Chemical Biology and Molecular Medicine Program, H. Lee Moffitt Cancer Center & Research Institute, 12902 USF Magnolia Drive, Tampa, FL 33612 USA

**Keywords:** Cancer, Cell biology, Chemical biology, Computational biology and bioinformatics, Drug discovery, Genetics, Immunology, Molecular biology, Stem cells, Diseases, Medical research, Oncology

## Abstract

A subset of hepatocellular carcinoma (HCC) overexpresses the chromosome 19 miRNA cluster (C19MC) and is associated with an undifferentiated phenotype marked by overexpression of cancer testis antigens (CTAs) including anti-apoptotic melanoma-A antigens (MAGEAs). However, the regulation of C19MC miRNA and MAGEA expression in HCCs are not understood. Here we show that, C19MC overexpression is tightly linked to a sub-set of HCCs with transcription-incompetent p53. Using next-generation and Sanger sequencing we found that, p53 in Hep3B cells is impaired by TP53-FXR2 fusion, and that overexpression of the C19MC miRNA-520G in Hep3B cells promotes the expression of MAGEA-3, 6 and 12 mRNAs. Furthermore, overexpression of p53-R175H and p53-R273H mutants promote miR-520G and MAGEA RNA expression and cellular transformation. Moreover, IFN-γ co-operates with miR-520G to promote MAGEA expression. On the other hand, metals such as nickel and zinc promote miR-526B but not miR-520G, to result in the suppression of MAGEA mRNA expression, and evoke cell death through mitochondrial membrane depolarization. Therefore our study demonstrates that a MAGEA-promoting network involving miR-520G, p53-defects and IFN-γ that govern cellular transformation and cell survival pathways, but MAGEA expression and survival are counteracted by nickel and zinc combination.

## Introduction

Chromosome-19 micro-RNA cluster (C19MC) is a large miRNA cluster located at the ‘q’ arm of chromosome 19, band chr19q13.42 within a span of ~ 100kb^1^. C19MC includes 46 miRNAs and is typically expressed only in the placenta among adult tissues^2,3^. Overexpression of C19MC miRNAs has been implicated in multiple cancer types such as hepatocellular carcinoma^4,5^, undifferentiated embryonal sarcoma of the liver^6^, breast cancer^2^, embryonal tumors with multilayered rosettes (ETMRs)^7^, infantile hemangioma^8^, thyroid adenomas^9^, testicular germ cell tumors^10^, and parathyroid tumors^11^.

Hepatocellular carcinoma (HCC) is one of the deadliest cancer types among human cancers and it accounts for estimated 42,220 new cases, and 30,200 deaths in United States alone in 2018 (for Liver & intrahepatic bile duct)^12^. The specific functional outcome of C19MC miRNAs in HCC is not fully understood yet, but a report implicates the co-expression of C19MC miRNAs with cancer testis antigens (CTAs)^13^. However, whether C19MC miRNAs lead to the expression of CTAs is not known. Melanoma antigen-A family genes (MAGEAs) are a subset of CTAs that are often expressed in various cancers^13^, and play an anti-apoptotic role by counteracting the p53-dependant cell death program^14^.

Using human hepatocellular carcinoma (LIHC) patient data here we show that, C19MC overexpression is tightly linked to a large set of CTAs including melanoma antigens in patients who harbor transcription incompetent p53. Using a combination of next-generation sequencing and in vitro experiments we further elucidate the role of C19MC miRNAs in regulating MAGEA expression. We identify a novel TP53-FXR2 fusion in Hep3B cells and show that the C19MC miRNA-520G but not miR-519D or miR-526B promotes the expression of MAGEA-3, 6 and 12. Overexpression of p53-R175H or p53-R273H mutant also promotes miR-520G and MAGEA mRNA expression in Hep3B cells. Finally, we identified IFN-γ as a co-operating factor to miR-520G and metals such as nickel and zinc as antagonistic factors of MAGEA mRNA expression to regulate cell death. Thus our study demonstrates a striking co-regulatory network between C19MC miRNAs, MAGEAs, IFN-γ and p53 in HCC and validates the oncogenic role of C19MC in HCC.

## Results

### C19MC overexpression specifically marks hepatocellular carcinomas with CTA expression, and miR-520G promotes the expression of MAGEAs-3, 6, and 12

**Figure 1:**
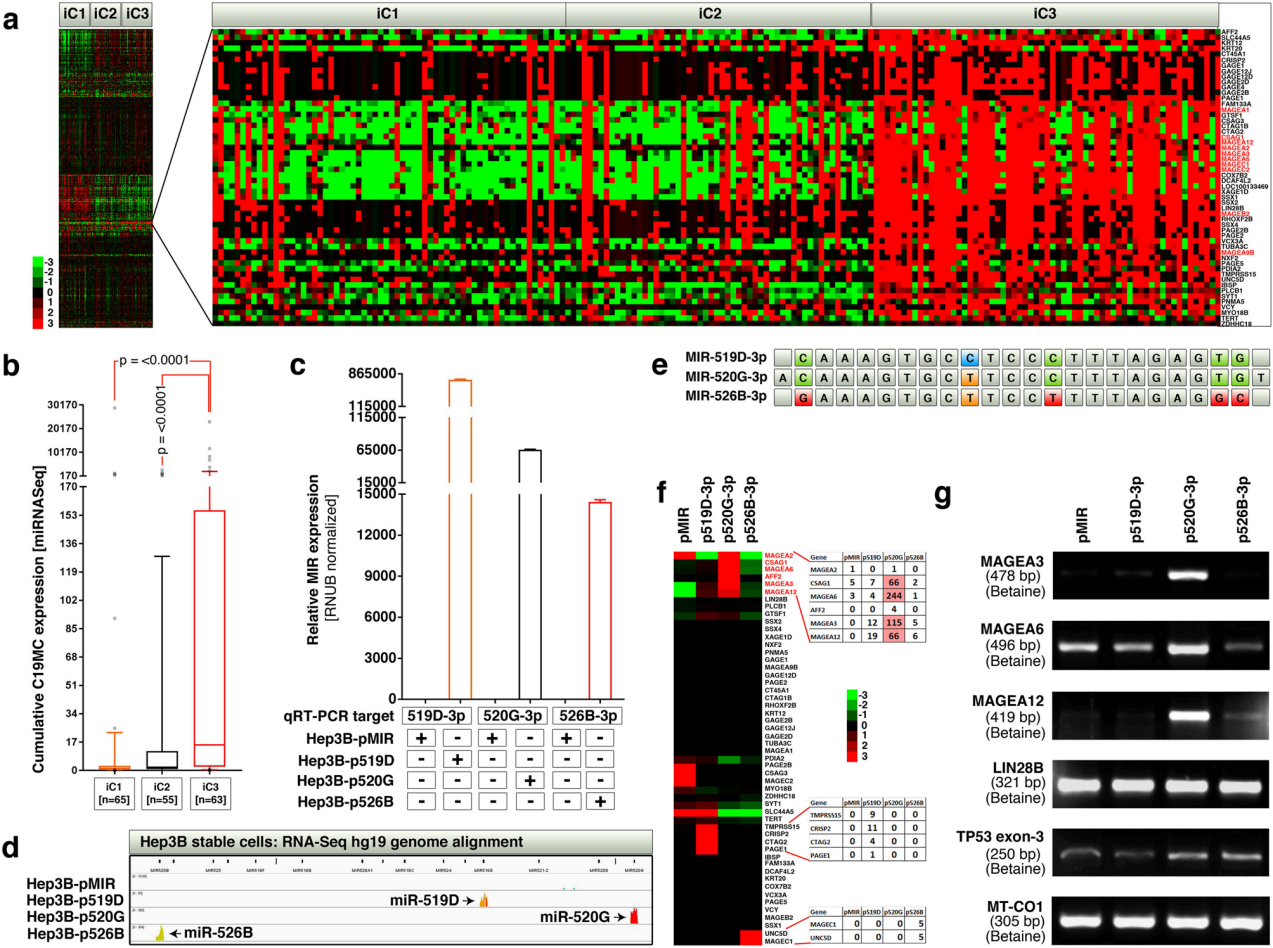
C19MC overexpression marks MAGEA expressing HCC iCluster-3 and miR-520G promotes the expression of MAGEAs-3, 6, and 12. (**a**) LIHC (HCC) iClusters showing overexpression of cancer testis antigens in iCluster-3. Melanoma antigens are shown in red font. (**b**) HCC iClusters showing overexpression of C19MC miRNAs in iCluster-3. Stably transfected Hep3B cells with C19MC miRNAs were confirmed by qRT-PCR (**c**) and RNA-seq (**d**). (**e**) Nucleotide differences between mature miR-519D, miR-520G, miR-526B. (**f**) RNA-seq heatmap of cancer testis antigens upregulated (in LIHC/HCC iCluster-3: panel-A) in Hep3B stable cells expressing miR-519D, miR-520G, miR-526B. Genes in red fonts are specific for miR-520G. (**g**) Reverse transcriptase PCR confirmation of MAGEA upregulation by miR-520G in Hep3B stable cells. LIN28B was selected in addition to MAGEAs.

To understand the expression pattern of C19MC miRNAs in human hepatocellular carcinoma in the context of CTAs, we re-integrated the Cancer Genome Atlas (TCGA) HCC-iCluster data set^15^ (miRNA-seq and RNA-seq). We found 54 genes that mainly included CTAs with melanoma antigens (MAGEAs) are expressed specifically in iCluster-3 (Fig. 1a) along with significantly high expression of C19MC miRNAs (Fig. 1b). To understand whether overexpression of C19MC miRNAs indeed regulate the expression of these CTAs, we generated stably transfected Hep3B cell lines overexpressing pMIR control vector, C19MC miRNAs 519D, 520G and 526B and verified the specific expression of miRNAs by quantitative real-time PCR (qRT-PCR) and RNA-seq (Fig. 1c-d). These three miRNAs show minor differences between their mature sequences (Fig. 1e). RNA-seq differential expression analysis of CTAs in these stable cells revealed that, although each miRNAs promoted specific set of CTAs, the read numbers are reliable only for miR-520G regulated MAGEA-3, 6, 12 and CSAG-1 (Fig. 1f). Of note, CSAG-1 gene shares a bivalent enhancer with the MAGEA12 gene (Supplementary Fig. 1) and both genes are upregulated by miR-520G, suggesting the bi-directional transcriptional activation of the enhancer. Therefore, MAGEA-3, 6, and 12 could be the prime promoted targets of miR-520G. We further confirmed the selective upregulation of MAGEA-3, 6 and 12 by miR-520G using RT-PCR along with LIN28B and TP53 (Fig. 1g). We included LIN28B and TP53 as test subjects because LIN28B is enriched in iCluster-3 along with CTAs (Fig. 1a) and is a major miRNA binding protein that intersects with mutant p53 signaling in cancer progression^16^, whereas p53 is a major mutated gene in HCC iCluster-3^15^. Of note, LIN28B was constitutively transcribed in Hep3B cells but was not influenced by any of the C19MC miRNAs tested (Fig. 1g). However, TP53 mRNA levels were moderately altered by miR-520G and 526B overexpression (Fig. 1g). Taken the iCluster-3 CTA expression data, and RNA-seq plus RT-PCR results on MAGEA expression together, it is conceivable that, C19MC miRNAs correlate with the expression of CTAs in iCluster-3 of HCCs and that individual C19MC miRNAs such as miR-520G promotes the expression of distinct subsets of CTAs such as MAGEA-3, 6, and 12, CSAG1 in the case of miR-520G.

### Hepatocellular carcinomas with high C19MC expression are incompetent for p53-dependent transcription: links to zinc and cell death

**Figure 2:**
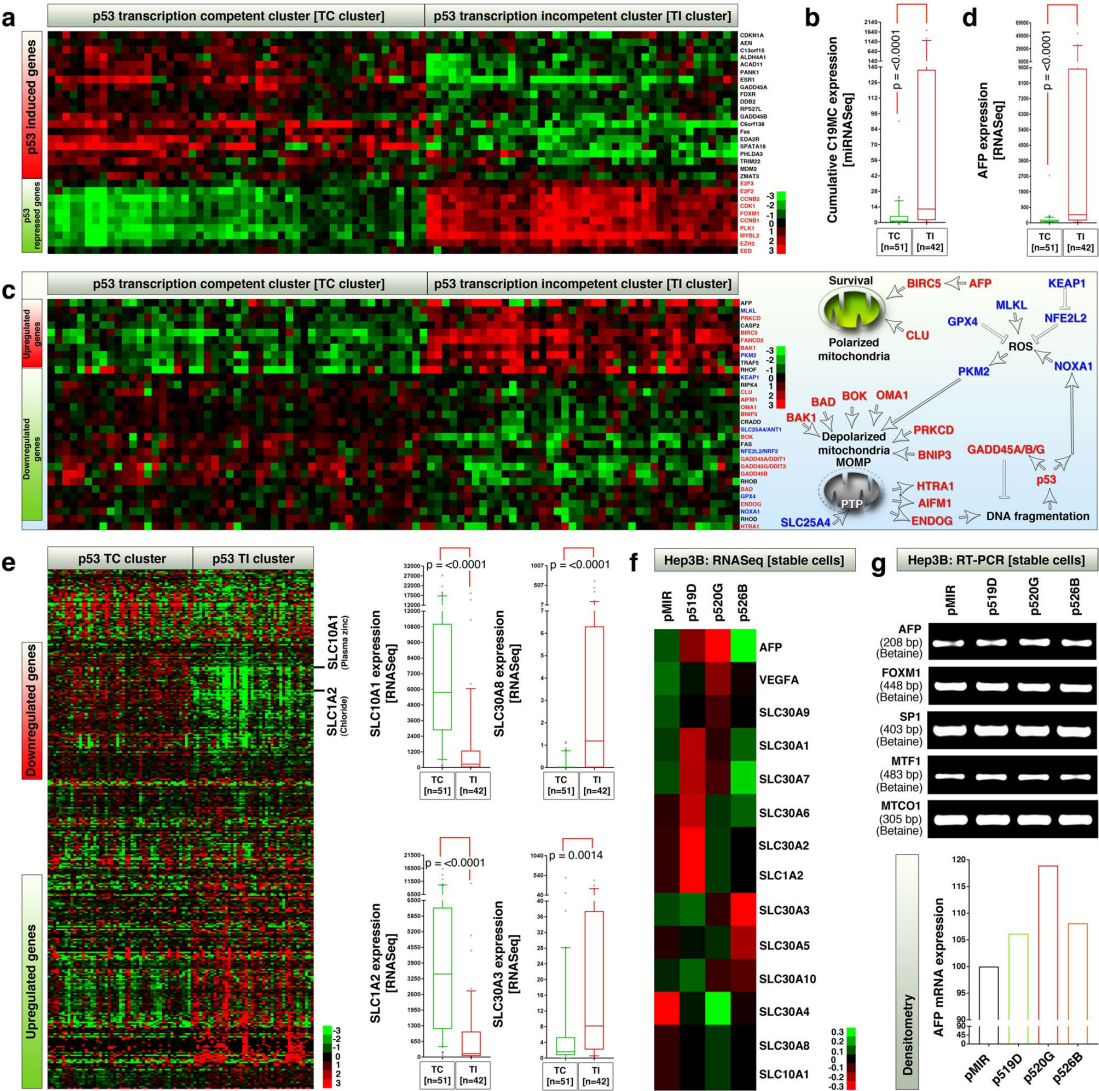
Overexpression of C19MC, AFP and zinc efflux pump RNAs are restricted to p53 transcription incompetent HCCs and miR-520G promotes AFP mRNA expression. (**a**) LIHC (HCC) classification of p53TC, p53TI tumors based on 10 p53-repressed genes and 20 p53-induced genes. Two large clusters that show clear differences were chosen. (**b**) HCCs showing overexpression of C19MC miRNAs in p53-TI cluster. (**c**) Heatmap of 31 genes showing differences between p53TC and p53TI clusters out of 200 cell death regulatory genes examined. (**d**) Overexpression of AFP in p53TI cluster (**e**) 324 SLC family gene heatmap showing a massive panel of SLC family members differ between p53TC and p53TI clusters. Major zinc-related SLCs are shown in box-whisker plots. (**f**) RNA-seq heatmap showing that, AFP, and zinc transporters/efflux pumps are promoted by C19MC miRNAs in stably transfected Hep3B cells. (**g**) RT-PCR showing C19MC miRNA regulation of AFP and AFP-transcription factors in Hep3B miRNA overexpressed Hep3B stable cells. Relative AFP mRNA expression quantified by densitometry is shown as bar graph in bottom panel.

In HCCs iCluster-3 harbors the highest rate of p53 defects^15^ and that melanoma-A antigens that are expressed in iCluster-3 are being shown to counteract p53-dependent transcription by targeting p53 to chromatin interaction^17^ and p53 transactivation to result in therapy resistance^14^. Therefore we examined whether C19MC expression is correlated to p53 functional status. For this purpose, we classified the TCGA iCluster dataset into p53 transcription competent (p53TC) and p53 transcription incompetent (p53TI) clusters [p53-TCTI dataset] (Fig. 2a) and examined the expression of C19MC in p53-TCTI dataset. We found that, C19MC is significantly overexpressed in p53TI cluster compared to p53TC cluster suggesting the involvement of p53 alteration in C19MC expression or function (Fig. 2b). Similarly, in 28 cancer cell lines that exhibit p53TC and p53TI signature (that includes cells from liver cancer and other cancer types) had relatively similar levels of C19MC miRNA expression, however, the MAGEA-3, MAGEA-6 and MAGEA-12 mRNAs were selectively upregulated in p53TI subset (Supplementary Fig. 2).

As expected, the p53TI cluster had overexpression of pro- and anti-cell death factors such as AFP, MLKL, PRKCD, CASP2, BIRC5, FANCD2, BAK1, PKM2, TRAF5 RHOF or downregulation of pro- and anti-cell death factors such as, CLU, AIFM1, OMA1, BNIP3, BOK, BAD, ENDOG, HTRA1, GADD45A, GADD45B and GADD45G (Fig. 2c). Notably, many of these gene products require, or bind to zinc for their function [examples: AFP, BIRC5/survivin, OMA1^18–20^], or their function is antagonized by zinc [Example: BAK1^21^]. Of particular interest, elevated AFP expression is a clinical biomarker for poor prognosis of HCCs^22^ and is a major zinc binding protein found in human plasma primarily expressed by liver^18^. AFP mRNA is significantly elevated in the p53TI cluster (Fig. 2d). Therefore we focused on the change in gene expression levels of entire solute carrier family proteins (SLC) which includes zinc transporters and zinc-efflux pumps^23^. In the p53TI cluster, many of the major zinc transporters (SLC1A2 and SLC10A1) were downregulated, whereas the zinc efflux pumps (SLC30A8 and SLC30A3) were upregulated (Fig. 2e), suggesting that the p53TI tumors do not prefer intracellular accumulation of zinc. RNA-seq data from C19MC miRNA stably overexpressed Hep3B cells revealed that, miR-520G, miR-519D and miR-526B promoted or downregulated distinct sets of SLCs and AFP (Fig. 2f). We further confirmed the upregulation of AFP mRNA by RT-PCR where miR-520G had considerable increase in AFP expression whereas the mRNAs of AFP transcription factors were largely unaffected except minor changes in metal sensing transcription factor MTF-1 (Fig. 2g). Taken together these results indicate that p53 transcriptional incompetence is associated with alterations in mRNAs of zinc regulated cell death factors, zinc transporters/efflux pumps, and that miR-520G mildly promotes AFP expression at RNA level.

### Hep3B cells express defective p53 mRNA and harbor *TP53-FXR2* fusion as a result of 76 kb focal deletion

**Figure 3:**
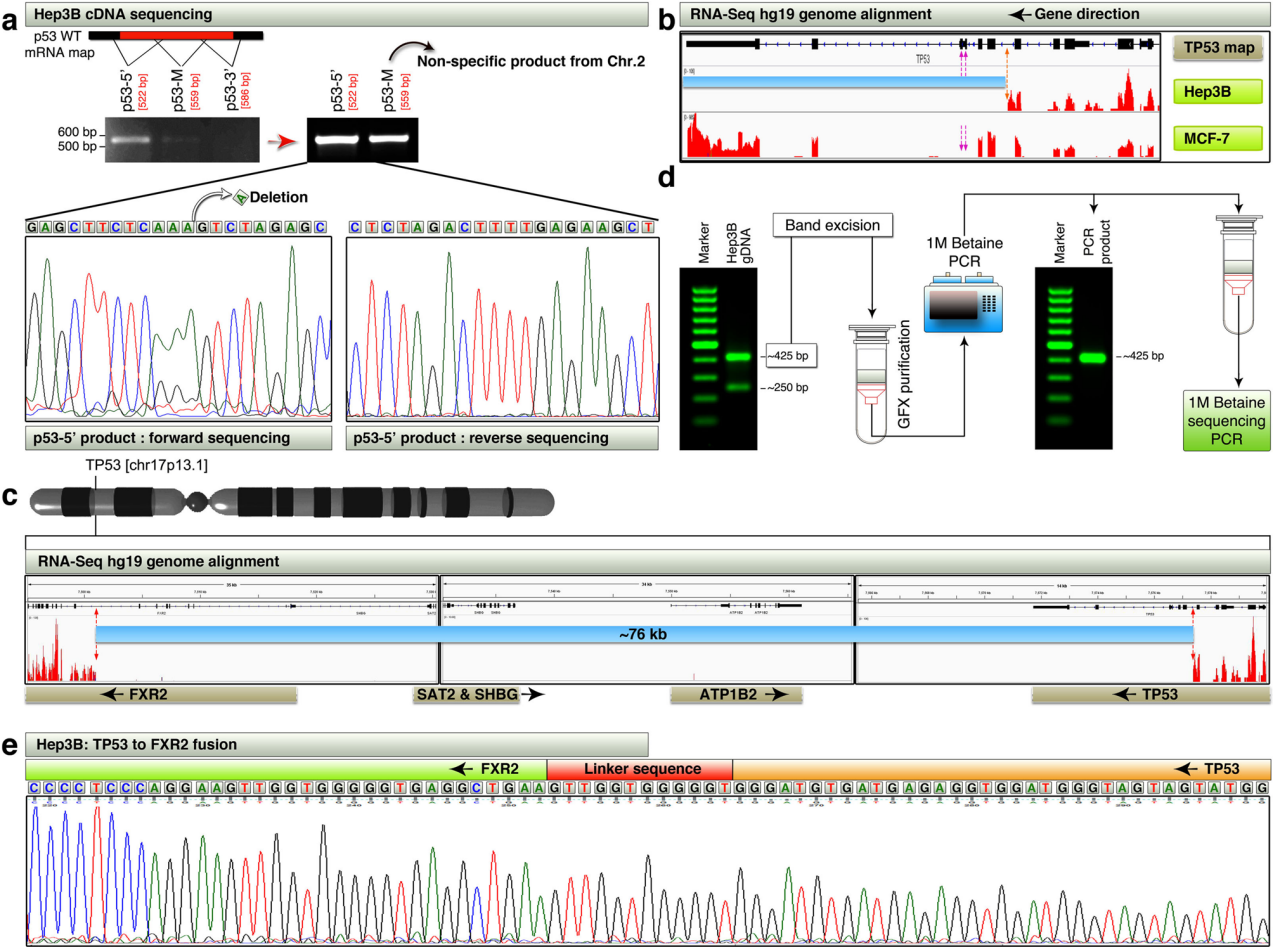
Hep3B cells express defective p53 mRNA and harbor TP53-FXR2 fusion as a result of 76 kb focal deletion. (**a**) RT-PCR and Sanger sequencing of p53 mRNA from Hep3B cDNA showing amplicons for 5’-end, and middle piece but not for 3’-end. The bands from left panel was re-amplified after GFX purification and shown on right panel. The p53-M turned out as a non-specific amplicon. Note the deletion of adenine in forward sequencing. (**b**) RNA-seq read mapping of Hep3B and MCF-7 cells to HG19 is visualized using IGV. Red dotted arrows in Hep3B show the potential deletion point and the blue bar shows the region of deletion (See panels c-e for more details). Purple dotted arrows show the exon skipping in MCF-7 cells. (**c**) Hep3B RNA-seq read mapping to HG19 showing lack of reads in 76 kb region from TP53 intron to FXR2 intron. Red dotted arrows show the potential deletion points within TP53 and FXR2 genes, and the blue bar shows the region of 76 kb deletion (See text for co-ordinate details). 3D chromosome-17 was created using Lightwave-3D (https://www.newtek.com/lightwave/) v11.6.3. (**d**) Amplification of potential TP53-FXR2 fusion product from Hep3B gDNA (Left gel panel) by PCR. GFX purification and re-amplification of single 425 bp amplicon (right gel panel), before subjecting the product for Sanger sequencing. (**e**) Sanger sequencing to confirm the TP53-FXR2 fusion in Hep3B cells. This fusion is also expressed at mRNA level (Data not shown). Note-1: the linker sequence may be introduced during the focal deletion and fusion process. Note-2: 1 M final conc. betaine is required to sequence this fragment as several attempts without betaine was failed to get clean sequence.

Given the fact that C19MC is overexpressed in p53TI HCC tumors, and p53 mutants can utilize zinc for gain-of-wild type-folding^24^, it becomes necessary to understand the nature of p53 defect in Hep3B cells. Hep3B is known to have defective p53^25^, but with different details reported on the nature of the defect (homozygous deletion of p53^26^, intragenic deletion^25^). Therefore we next examined the nature of *TP53* defect in Hep3B cells by exploring the cDNA using three sets of primers spanning from 5’ end to 3’ end through middle piece (M) (Fig. 3a). Only the p53-5’ was amplified (whereas the p53-M band turned out to be a misamplicon from chromosome-2: confirmed by Sanger sequencing) and paired-end sequenced to reveal a single nucleotide deletion, which is capable of shifting the reading frame. However, this nucleotide deletion was detected only in forward but not in reverse sequencing, suggesting that it could be a heterozygous point deletion (Fig. 3a). Because we failed to get amplicons from the middle piece and 3’end of p53 cDNA, we examined the genome aligned RNA-seq reads to see if it is due to deletion. While MCF-7 (a comparator cell line with intact *TP53*) RNA-seq revealed full-length exon reads for p53, the Hep3B cells had reads only up to the middle of the intron between exons 3 and 4, suggesting a deletion starting from this intronic region (Fig. 3b). Examination of the Hep3B RNA-seq reads upstream of this region (*TP53* is on negative strand) revealed that, the reads were lacking between ~ chr17: 7,501,000–7,577,371 [*TP53* intron (between exons 3 and 4) to *FXR2* intron (between exons 7 and 8); HG19] suggesting a focal 76 kb deletion from chromosome-17 (Fig. 3c). To confirm the deletion, we designed primers spanning from the suspected introns of *TP53* and *FXR2* and performed PCR using genomic DNA. The PCR resulted in a ~ 425 bp product and a ~ 250 bp product (Fig. 3d). We cut eluted 425 bp product and reamplified the band using same set of primers and made sure that it gives a single band (Fig. 3d). Sequencing of this band using 1 M betaine revealed that the *TP53* and *FXR2* were indeed fused, with a linker sequence of 12 nucleotides (GenBank accession: MN842296) (Fig. 3e). Taken together, these results demonstrated that Hep3B cells harbor a heterozygous single base pair frameshift capable deletion within the reading frame of *TP53* as well as a focal 76 kb deletion resulting in the fusion of the 5’-end of *TP53* with 3’-end of *FXR2*.

### Wild-type and mutant p53s promote miR-520G and MAGEA expression

**Figure 4:**
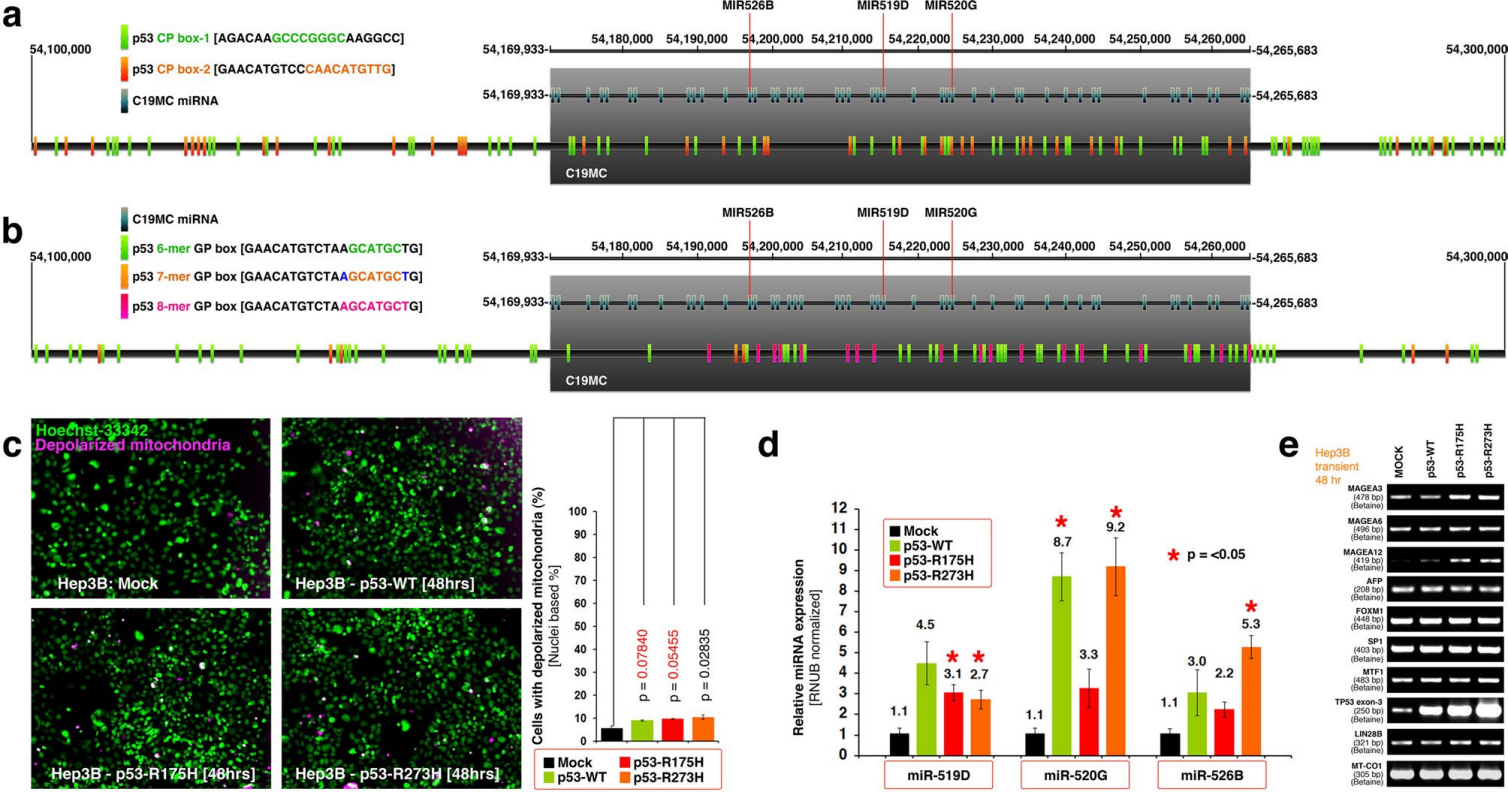
Wild-type and mutant p53s regulate miR-520G and melanoma antigen expression without inducing considerable cell death. (**a**) A map of CDKN1A gene’s p53 binding palindromic boxes-1 (green font/box) and 2 (orange font/box) [CP box] within C19MC (grey box) and surrounding landscapes. 6-mer-to-10-mer palindromic sequences were mapped to a 200 kb region enclosing C19MC. (**b**) A map of GADD45 gene’s p53 binding palindromic box (6-mer palindrome = green font/box), (8-mer palindrome = pink font/box) and (7-mer palindrome = orange font/box with 1 nucleotide match to 8-mer palindrome blue font) [GP box] within C19MC (grey box) and surrounding landscapes. (**c**) Transient transfection of p53-WT or mutant p53s does not induce considerable mitochondrial depolarization in Hep3B cells as evaluated by JC1 and Hoechst-33342 staining. The microscopy images were pseudo-colored as indicated. Purple corresponds to JC1 staining of depolarized mitochondria (MOMP). Also see Fig. 6D-E for more details. (**d**) Transient transfection of p53-WT or mutant p53s induce C19MC miRNA-520G expression in Hep3B cells as evaluated by qRT-PCR and is associated with induction/promotion of MAGEA mRNAs as evaluated by RT-PCR (panel-**e**).

Given the strong clinical association of p53TI tumors with C19MC overexpression and presence of TP53-FXR2 fusion in Hep3B cells, we sought to examine the effect of wild-type and mutant p53s in C19MC miRNA expression in Hep3B cells. DNA binding consensus of p53 usually harbors palindromic DNA binding sites of *CDKN1A* and *GADD45* genes that represent genome-wide and most prominent p53-binding consensuses^27,28^. The *CDKN1A* p53 binding sequence has two palindrome boxes within it. P53 *CDKN1A* palindromic box-1 (p53 CP box-1) has an 8-nucleotide palindrome GCCCGGGC which encompasses the 6-nucleotide palindrome of CCCGGG (Fig. 4a). The p53 CP box-2 has two 6-nucleotide palindromes of ACATGT, of which the second one is extended to a 10-nucleotide palindrome of CAACATGTTG (Fig. 4a). Both CP box-1 and CP box-2 palindromes are abundantly located within C19MC and its flanking regions (Fig. 4a). Similarly during DNA damage response p53 orchestrates transcription of a different set of genes using a *GADD45* consensus^29^. This consensus also has two *GADD45* palindromes (GP box) of which ACATGT is similar to *CDKN1A* consensus. The second *GADD45* palindrome is GCATGC and which can be extended to 8-nucleotide palindrome of AGCATGCT (Fig. 4b). While the GP box palindromes are also abundantly located within C19MC and its flanking regions, the 8-nucleotide GP boxes are located only within C19MC (Fig. 4b).

Considering the p53-binding palindromes within C19MC, alterations in zinc efflux pumps in p53-TI clusters, and the role of zinc in p53 folding and DNA binding [especially by the p53-R175H and p53-R273H metastasis promoting mutants^30–32^], we examined whether introduction of wild-type (p53-WT) or p53-R175H and p53-R273H mutants can promote C19MC miRNA transcription. Introduction of p53-WT, p53-R175H, or p53-R273H mutants did not promote considerable cell death by depolarization of mitochondria (Fig. 4c) except rare foci of cellular transformation (discussed below). However, p53-WT, p53-R175H, and p53-R273H all promoted individual C19MC miRNAs, most significantly with p53-WT or p53-R273H promoting miR-520G (Fig. 4d). Furthermore, p53-WT, p53-R175H, and p53-R273H promoted the expression of MAGEA12 and to a lesser extent the expression of MAGEA3 (Fig. 4e). The mRNA level expressions of AFP, or AFP transcription factors FOXM1, SP1, MTF1, or LIN28B did not change by the introduction of p53-WT, p53-R175H, or p53-R273H mutants in Hep3B cells (Fig. 4e). Taken together these results demonstrated that, wild-type and metastasis-promoting mutant p53s promote miR-520G and MAGEA expression.

### Nickel and zinc co-operate to drive non-apoptotic cell death associated with loss of MAGEA expression and promotion of miR-526B expression

**Figure 5:**
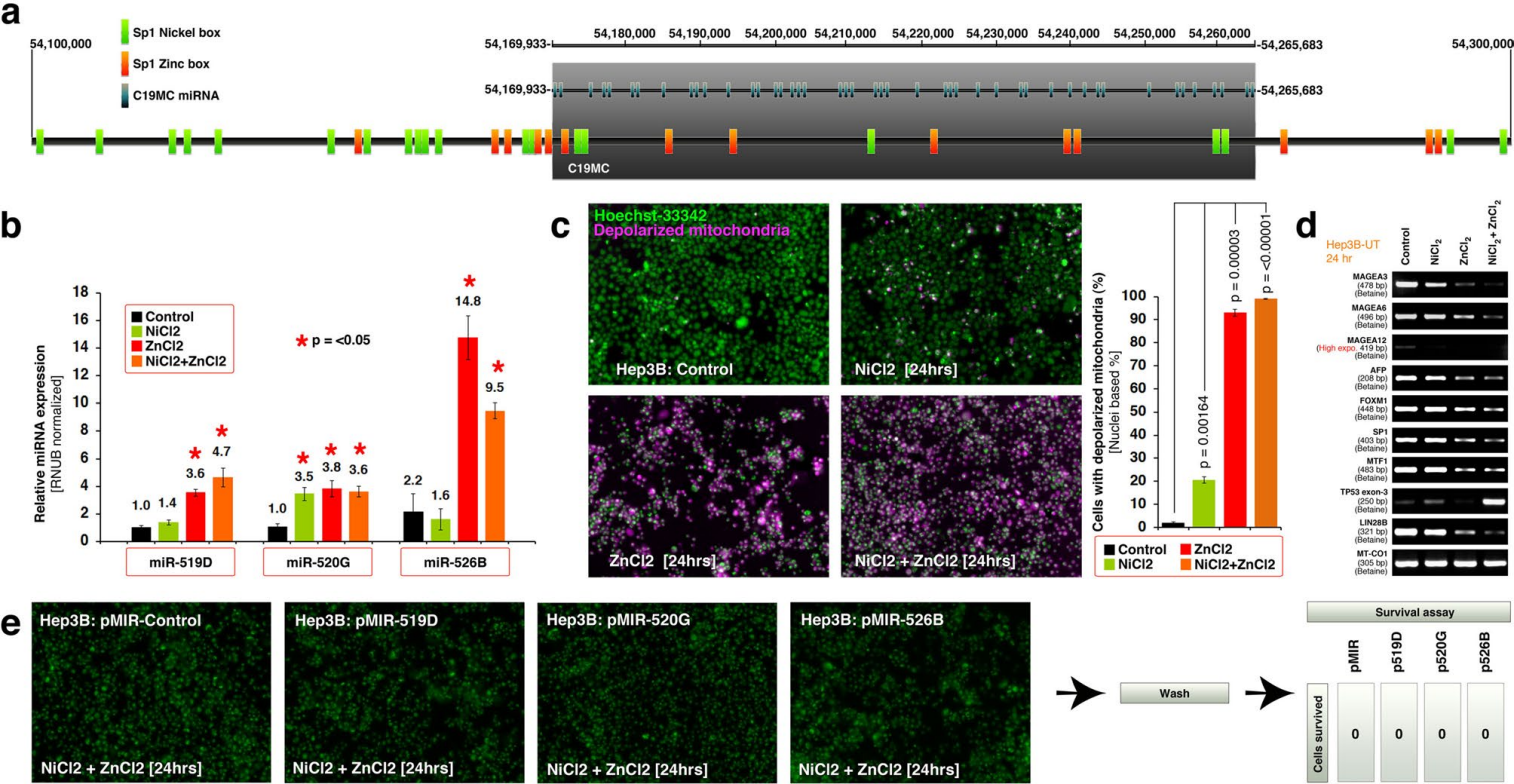
Nickel and zinc induce miR-526B and definite cell death in Hep3B cells. (**a**) A map of Sp1 zinc (orange) and nickel (green) dependent DNA binding boxes within C19MC (grey box) and surrounding landscapes. 6-mer-to-10-mer palindromic sequences were mapped to a 200 kb region enclosing C19MC. (**b–d**) Nickel and zinc induce C19MC miRNA-526B expression in Hep3B cells as evaluated by qRT-PCR (panel-**b**), massive mitochondrial depolarization as evaluated by JC1 and Hoechst-33342 staining (pseudo-colored: panel-**c**) and block the expressions of MAGEA and other mRNAs while promoting endogenous defective p53 as evaluated by RT-PCR (panel-**d**). (**e**) Nickel and zinc combination induces definite cell death in Hep3B cells that are stably overexpressed with C19MC miRNAs. After 24 h of treatment, the pyknotic cells were washed and plated to evaluate survival.

Zinc plays a prominent role in the function of p53-R175H, p53-R273H mutants and AFP. In the absence of the p53 transcriptional program, the AFP transcription factors Sp1 and MTF1 form transcriptionally active complex^33^. More intriguingly, Sp1 can switch DNA binding from one DNA element to other depending on the availability of zinc or nickel^34^ and can promote transcription even if the binding site is CpG methylated^35^. We found that, C19MC harbors multiple Sp1 binding sites with zinc or nickel binding specificity (Fig. 5a). A dose response in Hep3B cells using ZnCl_2_ revealed that 250 μM could induce 87.26% (± 3.78 SEM) pyknosis within 24 h (Supplementary Fig. 3). To understand the biology, we chose this dose for zinc as well as for nickel for further experiments. Zinc but not nickel significantly promoted the expression of miR-526B and did not have a considerable increase in miR-519D or miR-520G expression (Fig. 5b). Zinc induced depolarization of mitochondria in 93.0% (± 1.5 SEM) of the cells, whereas nickel induced only in 20.6% (± 1.4 SEM) of the cells (Fig. 5c). Interestingly, zinc and nickel downregulated MAGEA12, whereas zinc or zinc plus nickel combinations downregulated MAGEA3, MAGEA6, AFP, FOXM1, SP1, MTF1, and LIN28B (Fig. 5d) suggesting that, these metals inhibit transcription of these mRNAs. More importantly the zinc plus nickel combination dramatically promoted endogenous p53 (Fig. 5d). Of note, the zinc and nickel combination induced pyknoisis in all stable cells with pMIR, p519D, p520G and p526B and these pyknotic cells did not survive after washing and re-plating in complete growth medium (Fig. 5e) demonstrating the induction of definitive cell death.

**Figure 6:**
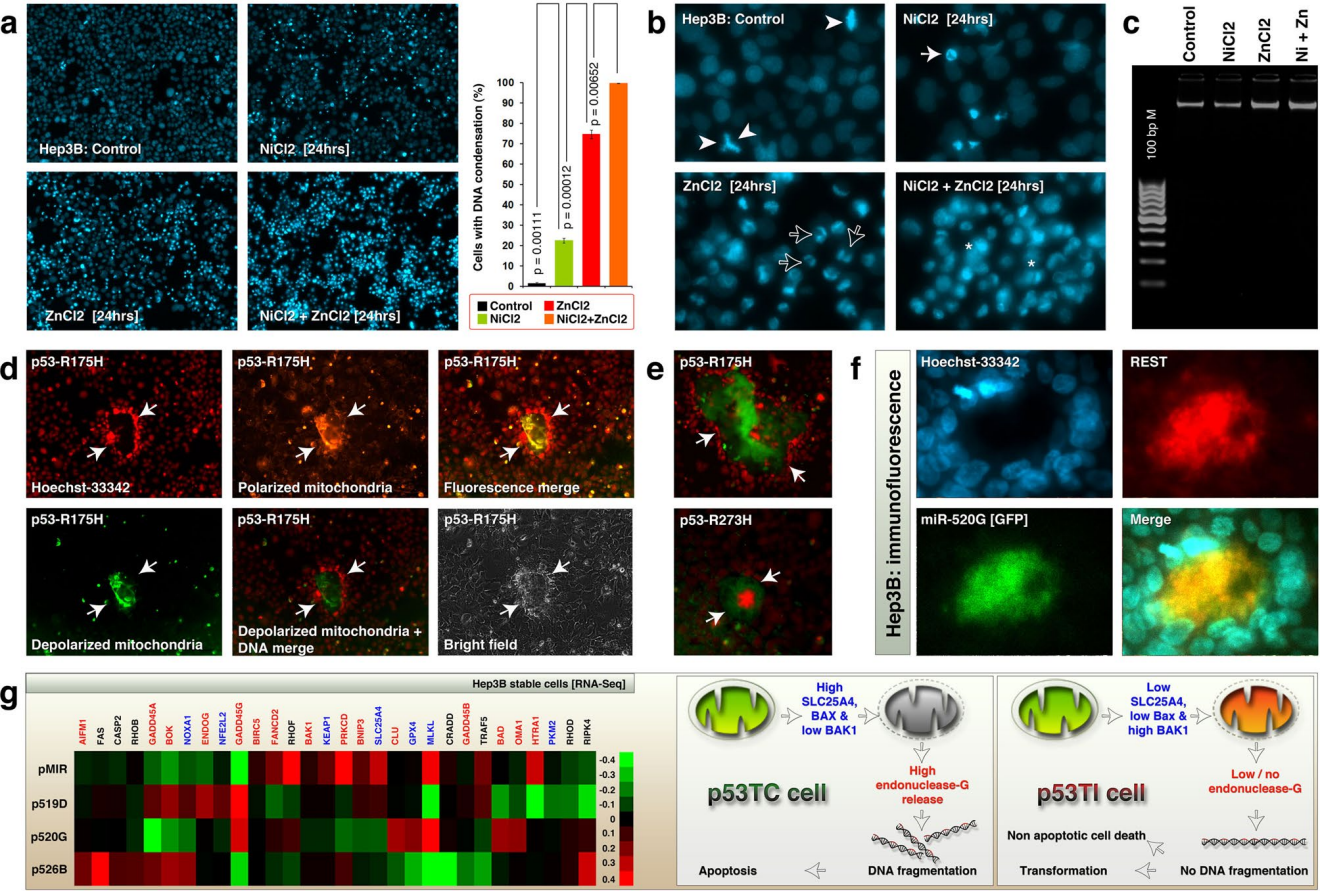
Nickel and zinc induce non-apoptotic cell death whereas mutant p53s and C19MC miRNAs induce transformation of Hep3B cells with depolarized mitochondria. (**a,b**) Nickel and zinc co-operatively induce DNA condensation in Hep3B cells that are stably overexpressed with C19MC miRNAs at 24 h of treatment (Panel-**a**). The mitotic DNA condensation (arrow heads) was carefully excluded from cell death related condensation (white and hollow arrows). Note the diffuse cytoplasmic DNA in combination treatment (*) [Panel-**b**]. (**c**) Neither nickel nor zinc or their combination induced DNA fragmentation at 24 h of treatment. Equal quantities of gDNA were run on 2% agarose gel. (**d,e**) Transient transfections of mutant p53s induce transformation (arrows) of Hep3B cells with mitochondrial depolarization at 48 h, as evaluated by JC1 and Hoechst-33342 staining. The microscopy images were pseudo-colored as indicated. Green corresponds to JC1 staining of depolarized mitochondria (MOMP). Red corresponds to DNA in DAPI channel. (**f**) REST immunofluorescence (Red) analysis of miR-520G (GFP) stable Hep3B cells showing cellular transformation which is surrounded by Hoechst-33342 positive (Blue) nuclei. Note: miRs-519D and 526B also stimulate similar transformation (Data not shown). (**g**) Evaluation of 30 cell death regulators that shown differential expression between p53TCTI dataset (Fig. 2c) in RNA-seq data of Hep3B cells stably transfected with C19MC miRNAs (Heatmap) and potential mitochondrial depolarization alteration that may explain lack of DNA fragmentation in Fig. 6 panel-c. Right panel: Schematic representing the differences in endonuclease-G release between p53TC and p53TI cells. The DNA was created using Lightwave-3D (https://www.newtek.com/lightwave/) v11.6.3. Rest of the image was created and composited in Adobe Photoshop CS5 (https://www.adobe.com/products/photoshopfamily/) v 12.0 × 64.

We next examined whether Hep3B cells undergo DNA condensation, a characteristic feature of multiple (but not all) cell death modalities. Nickel induced DNA condensation in 22.38% (± 1.2 SEM), zinc induced 74.65% (± 2.1 SEM), and the combination of nickel and zinc induced 99.59% (± 0.1 SEM), of Hep3B cells demonstrating the cell death promotion of zinc by nickel (Fig. 6a). In this experiment we carefully excluded mitotic chromosome condensation from cell death related DNA condensation as shown in Fig. 6b. Although the DNA was condensed during cell death it was not complete DNA condensation as in apoptotic bodies instead, diffused cytoplasmic DNA was also detected in zinc plus nickel combination (Fig. 6b). Therefore, we examined whether these cells undergo DNA fragmentation. Neither nickel nor zinc or their combination induced DNA fragmentation in Hep3B cells demonstrating the progression of non-apoptotic cell death (Fig. 6c).

Mutant p53 (R175H or R273H) transfected Hep3B cells exhibited rare transformed foci with massive accumulation of both depolarized mitochondria, polarized mitochondria and the foci were often but not always surrounded by nuclei (Fig. 6d-e). Similar transformed foci were also observed in miR-520G (Fig. 6f), miR-519D and miR-526B (data not shown) stable cells. However, we did not detect such transformed spheres in p53-WT or pMIR transfected cells. The downregulation of ENDOG, [a nuclease that fragment DNA during apoptosis] and SLC25A4/ANT1 [which trigger proper apoptosis by releasing endonuclease-G through permeability transition pores (PTPs) in mitochondria] in p53TI cluster (Fig. 2c) and in Hep3B miRNA stable cells (Fig. 6g) might explain why the cells did not undergo DNA fragmentation during nickel and zinc combination induced cell death.

Taken together these results demonstrate that, nickel and zinc co-operate to drive mitochondria depolarization-dependent non-apoptotic cell death and a rare type of cellular transformation associated with loss of MAGEA expression and promotion of miR-526B expression.

### IFN-γ co-operates but IL-6 antagonizes with miR-520G to regulate MAGEA, FOXM1, MTF1 mRNA expressions

**Figure 7:**
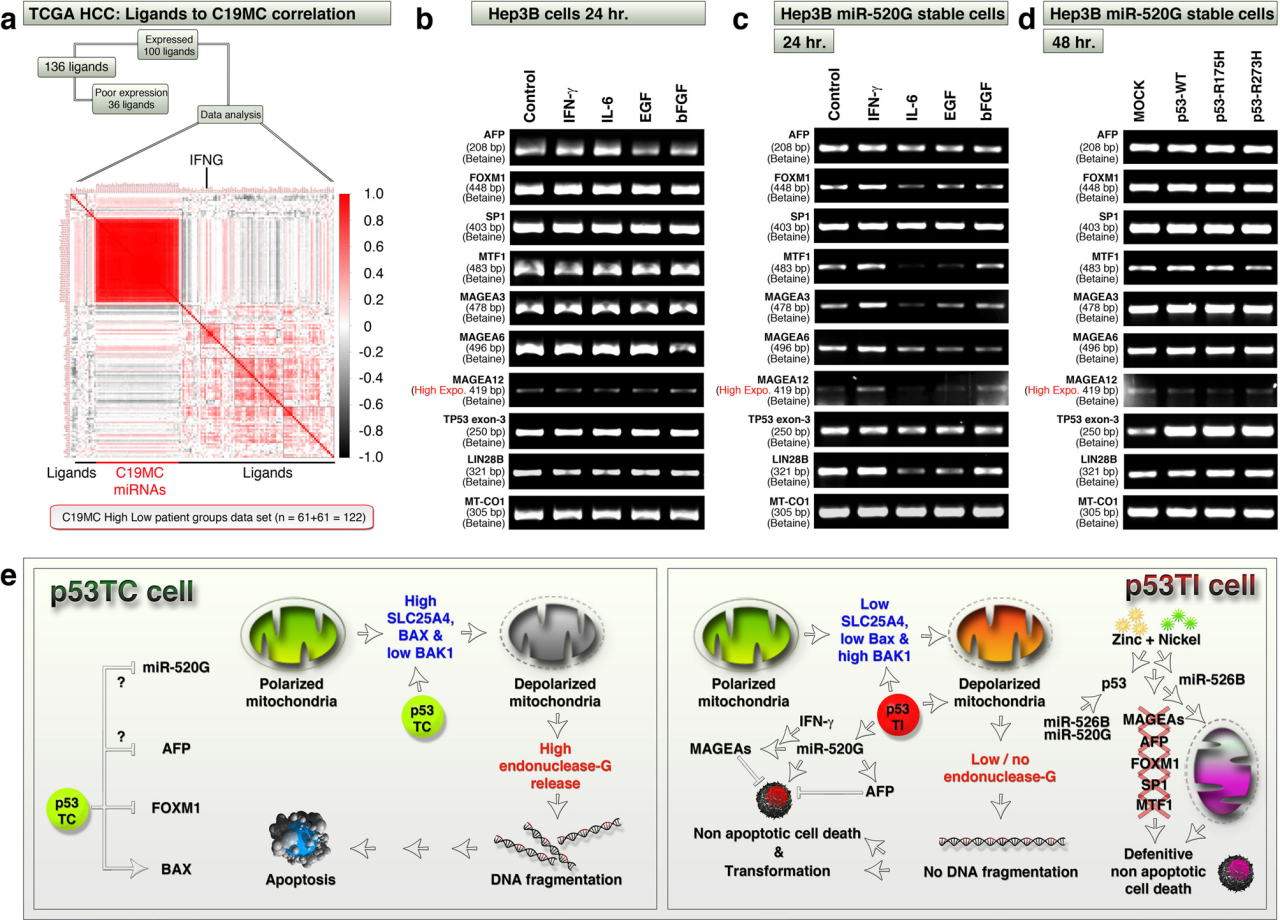
IFN-γ co-operates with miR-520G to upregulate MAGEAs, MTF-1 and FOXM1. (**a**) 100 expressing ligand mRNAs (cytokines, chemokines and so on) out of 136 from TCGA-LIHC data (RNA-seq data integrated to miRNA-seq from patients who had high versus low cumulative C19MC miRNA expression) were correlated to C19MC miRNAs to pick the top positively correlated IFN-γ mRNA. Insignificant and no correlations were color coded as white. (**b,c**) A panel of indicated gene mRNAs were examined for expression changes in response to indicated cytokines along with IFN-γ at 24 h time point in Hep3B untransfected cells (Panel-**b**), and C19MC miRNA-520G stably transfected cells (Panel-**c**). Note the differences between panels b and c. (**d**) Wild-type or mutant p53s does not co-operate with miR-520G to change the expression of any of the indicated genes considerably except MTF-1. (**e**) Schematic representing the cell death and transformation signaling differences between p53TC and p53TI cells. The apoptotic/non-apoptotic cells and DNA were created using Lightwave-3D (https://www.newtek.com/lightwave/) v11.6.3. Rest of the image was created and composited in Adobe Photoshop CS5 (https://www.adobe.com/products/photoshopfamily/) v 12.0 × 64.

To identify potential regulators of C19MC we subjected mRNAs of 100 ligands (that include cytokines and chemokines from RNA-seq of TCGA LIHC data integrated with miRNA-seq data, classified based on C19MC high and low groups) and 46 C19MC miRNAs for correlation analysis. We found that, while C19MC miRNAs themselves were highly correlated, *IFNG* mRNA is the candidate that correlated to most of the C19MC miRNAs (Fig. 7a). We tested the effect of IFN-γ in C19MC expression and MAGEAs expression in Hep3B cells along with other cytokines such as IL-6, EGF and bFGF. Recombinant IFN-γ neither promoted C19MC expression (as evaluated by qRT-PCR for miRs-519D, 520G and 526B: data not shown) nor promoted MAGEAs in Hep3B cells (Fig. 7b). However, in miR-520G stable Hep3B cells, IFN-γ promoted the expression of MAGEA-3, 6 and 12 along with FOXM1 and MTF1 mRNAs (Fig. 7c). Interestingly, IL-6 downregulated MAGEA-3, 6, 12, FOXM1, MTF1 and LIN28B mRNAs in miR-520G stable Hep3B cells, but could not do so in Hep3B parental cells (Fig. 7b-c). Furthermore, although mutant p53s and p53-WT promoted MAGEAs (Fig. 4e), these p53s did not promote MAGEAs in miR-520G stable cells (Fig. 7d). Taken together, these data demonstrate that, IFN-γ plays a co-operative role whereas IL-6 plays an antagonistic role with miR-520G in Hep3B cells to regulate MAGEA, FOXM1, MTF1 mRNA expressions.

## Discussion

The chromosome 19 microRNA cluster is overexpressed in a subset of hepatocellular carcinoma marked by the expression of cancer testis antigens (CTAs), including MAGEAs^13^. However, the regulation and function of C19MC miRNAs in HCC are not well known. We for the first time demonstrate that C19MC miRNA-520G promotes the expression of MAGEA-3, 6 and 12 in p53-defective cells and that C19MC miRNAs are overexpressed in p53 transcriptionally incompetent HCC tumors. Defects in p53 transcription can result in impaired apoptosis and enhanced cellular transformation^36,37^ and MAGEAs are known to inhibit p53 and p21 (a p53 transcriptional target) -induced apoptosis^38^. Although p53 is known to have defects in Hep3B cells, the exact nature of p53 defect in Hep3B cells was not known. We for the first time identify a novel *TP53-FXR2* fusion in Hep3B cells, which can potentially result in defective nuclear translocation and thus transcription^39^. Therefore, promotion of MAGEA-3, 6 and 12 in p53 defective Hep3B cells by miR-520G overexpression (Fig. 1g) reflects the MAGEA expression pattern of 28 cancer cell line p53TCTI signature (Supplementary Fig. 2). However, further analysis on MAGEA-3, 6 and 12 promoter and enhancers are necessary to understand the transcriptional regulators involved. In the context of CTAs, we have identified the regulation of MYO18B mRNA (another cancer testis antigen) by miR-520G in Hep3B cells^40^, which further supports the notion that the p53 defective background is associated with CTA expression through C19MC miRNAs. We additionally show that nickel and zinc can differentially regulate C19MC miRNA expression and when combined, the combination abrogates MAGEA expression and promotes definitive non-apoptotic cell death (Fig. 5e). Thus, our study delineates a striking co-regulatory network between C19MC, p53 transcriptional defects, and MAGEAs that is disrupted by metal ions zinc and nickel in HCC.

Cells can initiate blebbishield emergency program to undergo cellular transformation and survival, after the commitment of morphological and biochemical apoptosis ^41–54^. Mitochondrial depolarization can abrogate survival in the context of the blebbishield emergency program^47,52^, however, here we for the first time show that mutant p53-induced mitochondrial depolarization leads to cellular transformation with massive accumulation of depolarized mitochondria (Fig. 6d-e). However, cells undergoing pyknosis in response to nickel plus zinc combination could not survive or undergo transformation (Fig. 5e) and could not maintain mRNAs of MAGEAs, AFP, FOXM1, SP1, MTF1 and LIN28B suggesting the expression of C19MC miRNAs in deciding the survival or death of cancer cells that are defective in the p53 transcriptional program. MAGEAs play an anti-apoptotic role by counteracting the p53-dependant cell death program^14^ and therefore the regulation of MAGEAs at RNA level by C19MC miRNAs is a vital finding that sheds more light on the oncogenic role of C19MC. The overall signaling pathway in the context of C19MC miRNAs, mitochondrial depolarization, DNA fragmentation and cellular transformation is depicted in Fig. 7e.

In summary, we demonstrate an intimate co-regulatory network between C19MC, p53, and MAGEAs in HCC, where C19MC miRNA overexpression and p53 deficiency modulate MAGEA expression, cellular transformation and cell survival pathways.

## Materials and methods

### The Cancer Genome Atlas (TCGA) and iCluster details

TCGA miRNASeq, and RNASeq, data were from (https://gdac.broadinstitute.org/) and a patient data sub-set was selected from TCGA dataset based on the patient IDs of integrated clusters (iC1 + iC2 + iC3 = 183 samples) which was based on the expression of 528 signature genes (200 + 128 + 200 genes from iC1, iC2 and iC3 respectively) used to generate the prediction model as described previously^40^ in Figure S3A of reference^15^. The TCGA IDs and gene list of iClusters were kindly provided by Dr. Lee, Ju-Seog (UT MD Anderson Cancer Center, Houston, TX, USA), Dr. Ronglai Shen (Memorial Sloan Kettering Cancer Center, New York, NY, USA), Dr. David Wheeler (Baylor College of Medicine, Houston, TX, USA) and Dr. Lewis R. Roberts (Mayo Clinic, Rochester, MN, USA). This 183 patient dataset is referred to as HCC-iCluster data set which includes primary tumor samples of HCC patients who did not received any therapy, staged as per American Joint Committee on Cancer (AJCC) guidelines, subjected to pathology quality control using H&E slides as per previously published study^55^. The results in this study are in part based upon data generated by the TCGA Research Network: https://www.cancer.gov/tcga.

### Correlation plots, scripts, color code and statistical significance

Correlation plot to screen mRNAs of ligands that correlate with cumulative C19MC miRNA expression was generated using R package ‘corrplot’ 0.84 (was built under R version 3.4.4) by using the scripts as described previously^40^ but with minor modifications. Script: > cor(); > mat <—cor(); > corrplot(mat, order = "hclust", addrect = 6, method = "color"); > col1 <—colorRampPalette(c("black", "white", "red")); > corrplot(mat, order = "hclust", addrect = 6, method = "color", col = col1(100)), where addrect = 6 was optional; red = positive correlation; black = negative correlation. LIHC RNA-seq integrated to miRNA-seq data was classified into C19MC high versus low and the 61 high and 61 low samples (please see supplementary methods and supplementary table-3 for C19MC based group characteristics) were log transformed to the base of 10 before generating the matrix table in R. The insignificant correlations were coded white and thus white indicates either correlation value = 0 or insignificant.

### Cell lines, DNA fingerprinting, plasmids and stable/transient transfections

Human Hep3B cells (purchased from ATCC # HB-8064), and MCF-7 cells were cultured as described previously^40^. Briefly, cells were cultured in MEM containing L-Glutamine and Sodium bicarbonate (Sigma #M4655), with 10% FBS (Sigma#F0926), vitamins (Gibco Life Technologies #11120052), sodium pyruvate (Gibco Life Technologies #11360070), non-essential amino acids (Gibco Life Technologies #11140050), and penicillin–streptomycin (Gibco Life Technologies #15140122). DNA was isolated using QIAamp DNA mini kit (Qiagen # 51304) and the DNA were subjected to STR fingerprinting as per institutional/lab standards. The cells were then expanded, and frozen. Fresh vials were used after every 6 months or after ~ 25 passages. The cells in culture were tested for mycoplasma periodically using MycoAlert Kit (Lonza). The cell lines used in this manuscript were maintained from January 2018 to May 2019.

Glycerol stocks of mammalian expression vectors such as pMIR-CMV, pMIR-CMV-519D (CR215546), pMIR-CMV-520G (CR215781), pMIR-CMV-526B (CR215142) were purchased from Vigene Biosciences (Rockville, MD USA). Plasmids were isolated using Qiagen MIDI prep kit (#12143).

Hep3B cells were stably transfected using plasmids and Lipofectamine 2000 (Life Technologies # 11668019) and selected using 4 μg/ml puromycin (Invitrogen # A1113803) for 2 months. The cells were then sorted for GFP positive cells, expanded and frozen. For transient transfections, 1 μg plasmid DNA/10 cm dish were used with lipofectamine for 12–14 h in complete MEM, the media was washed off, and the cells were then collected at the 48 h time point (from the time of addition of DNA + lipofectamine complex to cells).

### Quantitative real-time PCRs [qRT-PCRs]

Quantitative RT-PCRs were done as described previously^40^. Briefly, RNAs were isolated using miRNeasy Mini Kit (Qiagen #217004, Germantown, MD, USA), quantified using Nanodrop, and 250 ng RNAs were subjected to cDNA synthesis using Multiscribe reverse transcriptase with RNAse inhibitor, 10 × buffer, dNTPs, (ABI, Cat # 4366596) and RT TaqMan Primers (RNU6B Control Assay: Assay ID: 001093 (Cat # 4427975), hsa-miR-519d [For 3p]: Assay ID: 002403 (Cat # 4427975), hsa-miR-520 g-3p : Assay ID: 001121 (Cat # 4427975), hsa-miR-526b-3p: Assay ID: 002383 (Cat # 4427975). Then the cDNAs were subjected to real-time PCR reactions using corresponding primers with probes and Taqman master mix in triplicates. Comparative Ct (Δ ΔCt) method was used to calculate the relative expression of miRNAs after normalizing with RNU6B values. The results were then plotted using Graphpad Prism software (v7.04; La Jolla, CA, USA).

### RNA-seq and IGV visualization

RNAs were isolated using miRNeasy Mini Kit (Qiagen #217004, Germantown, MD, USA), with an on-column RNAse free DNAse (Qiagen # 79254) digestion as per manufacturer’s protocol. RNA-seq was performed using the NuGen Ovation RNA-seq FFPE System (PN 7150–08) to prepare the libraries and were run on the Illumina NextSeq 500 with a 76-base paired-end read. The adapter reads were detected using BBMerge (v37.88)^56^ and trimmed using Cutadapt (v1.8.1)^57^. The adapter sequences were: AGATCGGAAGAGCACACGTCTGAACTCCAGTCA and AGATCGGAAGAGCGTCGTGTAGGGAAAGAGTGT. Trimming was part of the standard Illumina BaseSpace FASTQ Generation app that runs automatically after sequencing run. Processed raw reads were then aligned to human genome (build: hg19) using STAR (v2.5.3a)^58^. Gene expression was evaluated as read count at gene level with HTSeq (v0.6.1)^59^ and Gencode gene model v28. Gene expression data were then normalized and differential expression was evaluated using DEseq2^60^. The indexed reads were visualized using IGV v2.4.14 using HG19 genome.

### TP53 mutation analysis & Sanger sequencing and identification of TP53 to FXR2 fusion

#### Mutation analysis

Total RNA was isolated from Hep3B cells as described above, and cDNAs from RNAs were generated using 1.5 M betaine as described in Reverse transcriptase PCRs section (Supplementary methods). The p53 cDNA was amplified using primers mapping to 5’ end (nt. 1–522), middle piece (M: nt. 496–1054), and 3’ end (nt. 961–1546) of coding sequence where the TP53’s 5’ and 3’ end primers overlapping with UTRs. The nucleotide positions were based on the GenBank RNA sequence of NM_000546.5. The primer sequences were included in Supplementary table-2. The PCR products obtained were gel purified using GFX columns (illustra GFX PCR DNA and Gel Band Purification Kit, GE Healthcare # 45001489) and subjected to paired-end Sanger sequencing.

#### Identification of TP53 to FXR2 fusion in Hep3B cells

Based on RNA-seq IGV reads primers were designed to amplify TP53 to FXR2 focal deletion point. The 53INTR reverse complement primer mapping to TP53 intron (HG19: Chr17: 7,577,480) and the FXINTF sense primer mapping to FXR2 intron (HG19: Chr17: 7,500,880) were used to amplify the fusion sequence of ~ 425 bp using Hep3B genomic DNA or cDNA with 1 M betaine. The primer sequences were included in Supplementary table-2. The resultant product from gDNA was gel excised, GFX purified as described above, and re-amplified to exclude the ~ 250 bp product that resulted in the initial PCR reaction. The 425 bp product was subjected to Sanger sequencing with 1 M betaine. The fusion sequence is submitted to GenBank (Accession: MN842296).

### Sp1 (nickel and zinc box), p53 (CDKN1A and GADD45 palindrome box) sequence analysis in C19MC

A 200 kb chromosome 19 sequence (Chr19:54,100,000–54,300,000: HG19) encompassing 96 kb C19MC was mapped for Sp1 binding sites with preference for zinc and nickel as described previously^34^ and for palindromic sequences within p53 binding consensus of CDKN1A and GADD45 as described previously^27,29^. The distances between mapped sequences were converted to percentage to create a binding site map corresponding to HG19 co-ordinates.

### Statistical analyses

Statistical analyses were performed as described previously^2^. Briefly, frequency distribution 10–90 percentile type box-whisker plots and statistical analyses were done using Graphpad Prism software (v7.04; La Jolla, CA, USA). In box-whisker plots the outliers are aligned with 75% transparency. For patient group versus group statistical significance analysis (box-whisker plots), unpaired, non-parametric Mann–Whitney test was used. Throughout the study the p-value of 0.05 was considered significant and for frequency distribution box-whisker plots, p-values < 0.001 were considered as robust significance. The ‘n’ for TCGA data analysis were indicated in figures. For wet lab experiments, t-tests were performed using two-tailed distribution, two sample unequal variance. Error bars represent standard error of mean. For statistics on patient clinical characteristics in C19MC high versus low groups, TCGA cBioportal for Cancer Genomics (https://www.cbioportal.org/)^61,62^ was used. For clinical characteristics that do not have statistics in cBioportal, the TCGA clinical data from GDAC firehose (https://gdac.broadinstitute.org/) was manually curated by integrating C19MC high and low group patients with clinical dataset and the statistical significance was examined using unpaired, non-parametric Mann–Whitney test in Graphpad Prism software.

## Supplementary Information

Methods including C19MC-based grouping of HCC patient data & heatmap analysis, p53 transcription competent (TC) and transcription incompetent (TI) HCC sample clustering of TCGA iCluster sub-sets and CCLE cell lines, heatmaps for SLC family and cell death, immunofluorescence analysis & microscopy, mitochondria polarization status analyses, reverse transcriptase PCRs & primer sets, chromatin condensation and DNA fragmentation analyses were included in supplementary materials.

Supplementary Information.
