# Supplementary material for: Mutant p53s and chromosome 19 microRNA cluster overexpression regulate cancer testis antigen expression and cellular transformation in hepatocellular carcinoma
Source: Sci Rep. 2021 Jun 16;11:12673. doi: 10.1038/s41598-021-91924-7 (PMC8209049; doi:10.1038/s41598-021-91924-7)
Supplement: Supplementary file 1 — Supplementary Information. [file 41598_2021_91924_MOESM1_ESM.pdf]

Mutant p53s and Chromosome 19 microRNA Cluster Overexpression Regulate Cancer Testis Antigen Expression and Cellular Transformation in Hepatocellular Carcinoma

Goodwin G. Jinesh, Marco Napoli, Marian T. Smallin[1,2], Andrew Davis, Hayley D. Ackerman, Payal Raulji, Nicole Montey, Elsa R. Flores, and Andrew S. Brohl

Supplementary methods

C19MC-based grouping of HCC patient data and heatmap analysis

Gene expression by miRNASeq dataset of LIHC was processed to get cumulative miRNA expression of all 46 C19MC miRNA genes (*MIR498, MIR512-1, MIR512-2, MIR515-1, MIR515-2, MIR516A1, MIR516A2, MIR516B1, MIR516B2, MIR517A, MIR517-B, MIR517C, MIR518A1, MIR518A2, MIR518B, MIR518C, MIR518D, MIR518E, MIR518F, MIR519A1, MIR519A2, MIR519B, MIR519C, MIR519D, MIR519E, MIR520A, MIR520B, MIR520C, MIR520D, MIR520E, MIR520F, MIR520G, MIR520H, MIR521-1, MIR521-2, MIR522, MIR523, MIR524, MIR525, MIR526A1, MIR526A2, MIR526B, MIR527, MIR1283-1, MIR1283-2, and MIR1323*) and matched to the HCC-iCluster RNA-seq data set [n = total (183): iC (65); iC2 (55); iC3(63)] to get RmiDataset (RNASeq and miRNASeq integrated dataset). The clinical characteristics and statistical significances were included in supplementary table-3. The datasets were ordered based on iClusters 1-3 and were subjected to heatmap analysis using Cluster 3.0 (Filtering: SD=200, log transformed, genes and arrays centered; Hierarchical: Genes clustered, correlation centered, without array clustering, average linkage) and Java Treeview 1.1.6r4.

Transcription competent (TC) and Transcription incompetent (TI) p53 sample clustering of iCluster sub-set and heatmaps for SLC family and cell death

RmiDataset (described above) is subjected to heatmap analysis using 30 signature genes that represent p53 transcription competence (p53-induced and p53-repressed genes) [1] were subjected to cluster analysis (Filtering: SD=30, log transformed, genes and arrays centered; Hierarchical: Genes clustered-correlation centered, arrays clustered-correlation centered, average linkage) to generate heatmap and the two largest clusters that shown clear differences between p53-induced genes and p53-repressed genes were designated as p53 transcription competent (p53-TC) and p53 transcription incompetent (p53-TI) clusters. For the list of patient TCGA IDs of p53-TC and p53-TI clusters (p53-TCTI dataset) please refer Supplementary table-1.

The p53-TCTI dataset was further subjected to heatmap analysis using entire solute carrier family (SLC) of genes (n=324) or 200 genes related to cell death (includes: apoptosis, necrosis, necroptosis, ferroptosis, pyroptosis, and other forms of cell death[2]) (Filtering: %present >100, log transformed, genes and arrays centered; Hierarchical: Genes clustered-correlation centered, arrays clustered-correlation centered, average linkage).

Cancer Cell Line Encyclopedia (CCLE) and cell line p53TCTI dataset details

RNA-seq and miRNA-seq data of cancer cell lines were from Broad Institute CCLE database (https://portals.broadinstitute.org/ccle). The miRNA-seq data was integrated to RNA-seq data and cell lines that express similar levels of cumulative C19MC miRNAs were chosen and subjected to cluster analysis using 30 signature genes that represent p53 transcription competence (p53-induced and p53-repressed genes) [1]. Fourteen p53TC and 14 p53TI cell lines were chosen to examine the expression level of MAGEA-3, MAGEA-6 and MAGEA-12 using Graphpad Prism software (v7.04; La Jolla, CA, USA), and represented as 10-90% boxplots.

Immunofluorescence analysis and microscopy

Stable miR-520G GFP expressing cells were plated in 24 well plates at high density (200,000 cells/ml) and cultured for 48hrs with a media change at 24hrs. The cells were methanol fixed in -20°C for a period of 24-48hrs, washed with PBS, blocked for 30 minutes at room temperature with blocking buffer (1% BSA and 0.3% Triton-X100 in PBS). Then the cells were incubated overnight with REST/NRSF Antibody (F-3) Alexa Fluor-546 (Sc-374611, Santa Cruz Biotechnology, Dallas, TX, USA: Dilution 1:50) in blocking buffer. The cells were washed and incubated with Hoechst-33342 for 20 minutes, washed again before imaging using Zeiss Observer.Z1 microscope equipped with Axiocam 503 mono (Zeiss) camera. The individual channel images were pseudo-colored if indicated in figures/legends, merged and exported using ZEN 2.3 Pro software (Carl Zeiss Microscopy, GmbH, 2011, Blue edition). The final composite was done using Adobe Photoshop CS5 (Adobe Systems Inc., San Jose, CA, USA).

Mitochondria polarization status analyses

Cells in triplicates after indicated treatment/culture duration were added JC-1 dye (Cayman Chemicals #15003) and Hoechst-33342 (Cayman Chemicals #15547) as per manufacturer's instructions. Depolarized mitochondria, polarized mitochondria and DNA were imaged using green, red and blue channels respectively and pseudo-colored as indicated in figures/legends. The cells with depolarized mitochondria+ DNA condensation and total number of cells per frame were counted and the percentages of cells with depolarized mitochondria were calculated. Image processing was done as described under immunofluorescence section.

Chromatin condensation and DNA fragmentation analyses

Chromatin condensation assay:

Cells in triplicates after indicated treatment/culture duration were added 10 nM Hoechst-33342. DNA was imaged using blue channel. The cells with DNA condensation and total number of cells per frame were counted and the percentages of cells with DNA condensation were calculated. Image processing was done as described under immunofluorescence section.

DNA Fragmentation assay:

Cells were treated with 250 μM of $NiCl_2$, $ZnCl_2$ or their combination for 24 hrs. The cells were scrapped in existing media, pelleted, washed with PBS and subjected to DNA isolation using QIAamp DNA mini kit (Qiagen # 51304) as per manufacturer's protocol. The DNA was then resolved in 2% agarose gel in TAE buffer alongside of marker (GeneRuler 100 bp DNA Ladder: ThermoFisher Scientific # SM0243).

Reverse transcriptase PCRs

Total RNA was isolated using TRIZOL reagent (ThermoFisher Scientific #15596026, Waltham, MA, USA) as per manufacturer's instructions. 20 μl complementary DNA synthesis reactions were done using 1000 ng RNA and High-Capacity cDNA Reverse Transcription Kit (ABI # 4368814, Foster City, CA, USA) with 1.5M final concentration of betaine (from 5M stock: Sigma # B0300-1VL, St. Louis, MO, USA). The temperature conditions were, 25°C for 10m, 37°C for 120m and 85°C for 5m. The cDNAs were then diluted with 30 μl of nuclease free water and then 2.5 μl was used per PCR reaction. For PCR reactions 1M betaine (final conc.) was used along with regular PCR reaction components. The primer sequences were included in Supplementary table-2. All PCR reactions were standardized with a denaturing (95°C) time of 1 minute, annealing temperature of 60°C (30 seconds) and 1 minute of extension time (72°C),

with 34 cycles. The PCR reactions were run on 2% agarose gels with GeneRuler 100 bp DNA Ladder (ThermoFisher Scientific #SM0243). The gels were imaged using LI-COR Odyssey Fc imager (Lincoln, NE, USA).

Supplementary references

1 Cancer Genome Atlas Research Network. Electronic address wbe, Cancer Genome Atlas Research N. Comprehensive and Integrative Genomic Characterization of Hepatocellular Carcinoma. Cell 2017; 169: 1327-1341 e1323.

2 Galluzzi L, Vitale I, Aaronson SA, Abrams JM, Adam D, Agostinis P *et al*. Molecular mechanisms of cell death: recommendations of the Nomenclature Committee on Cell Death 2018. Cell Death Differ 2018; 25: 486-541.

Supplementary figure - 1

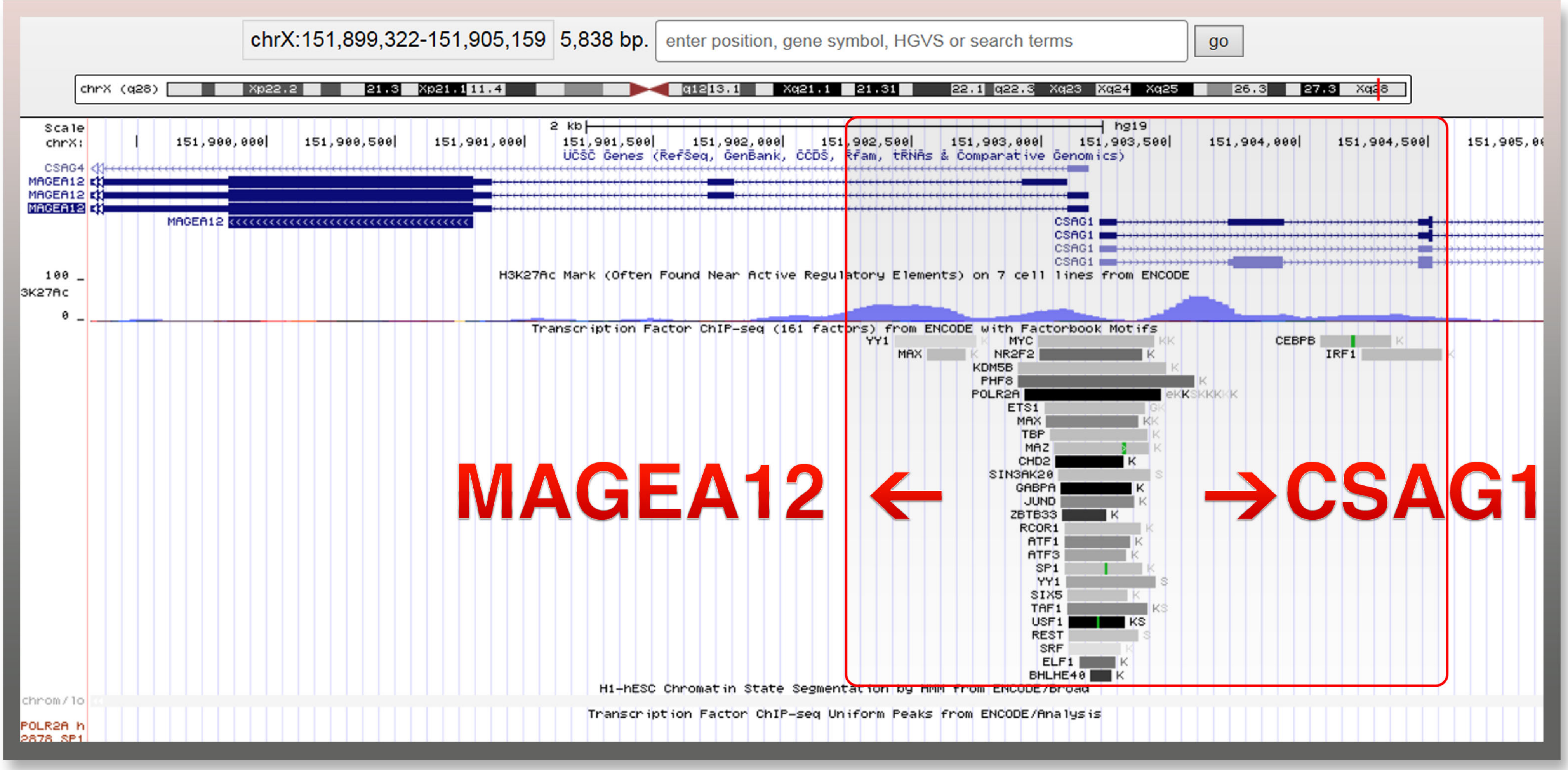

Supplementary figure - 2

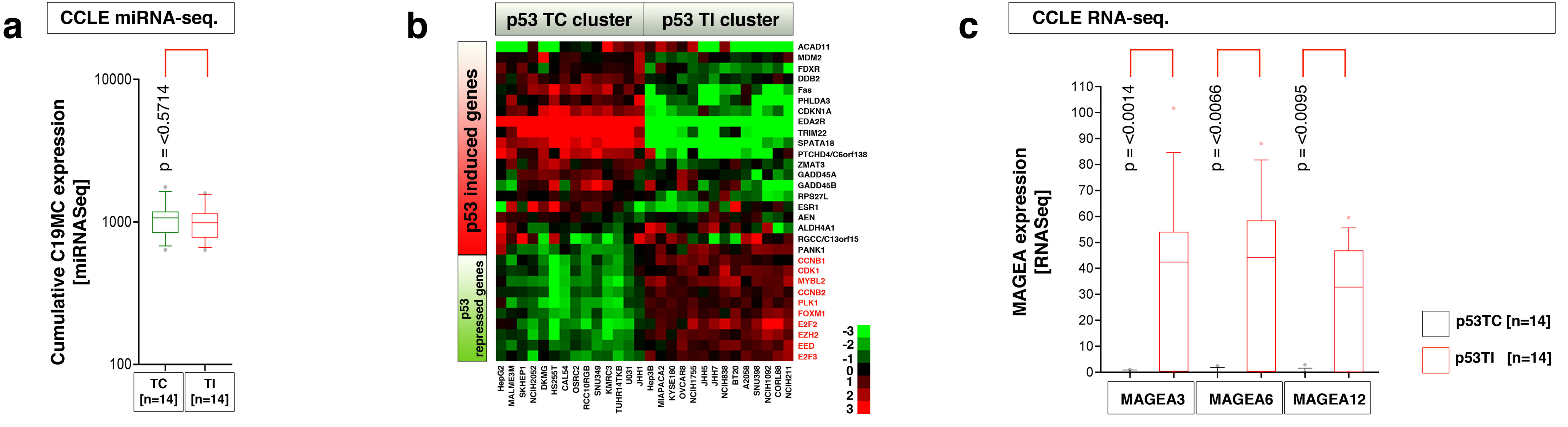

Supplementary figure - 3

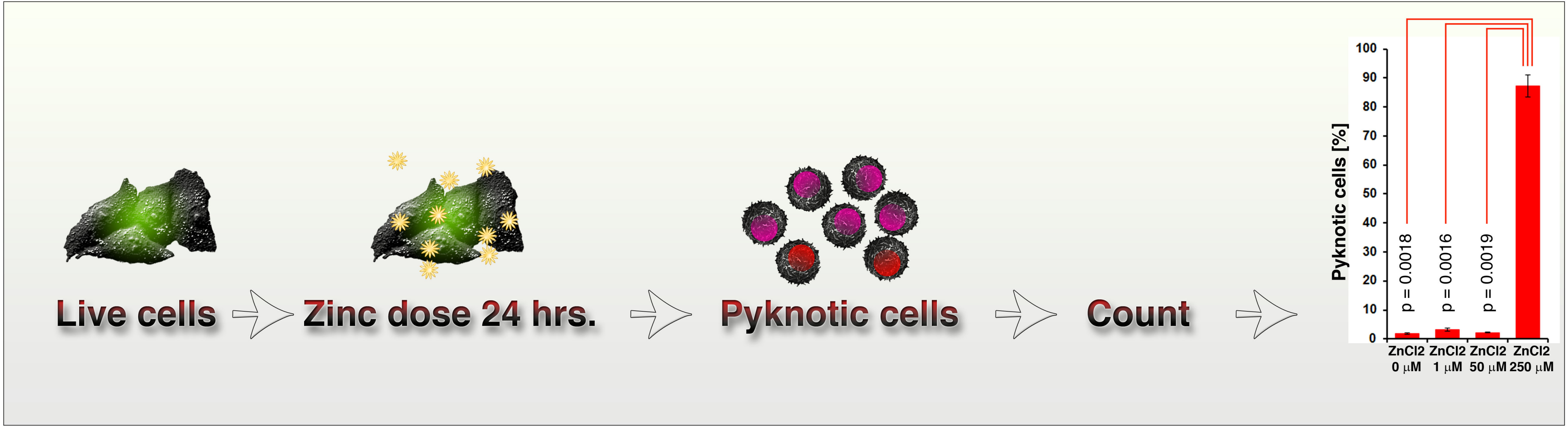

Supplementary figure-4

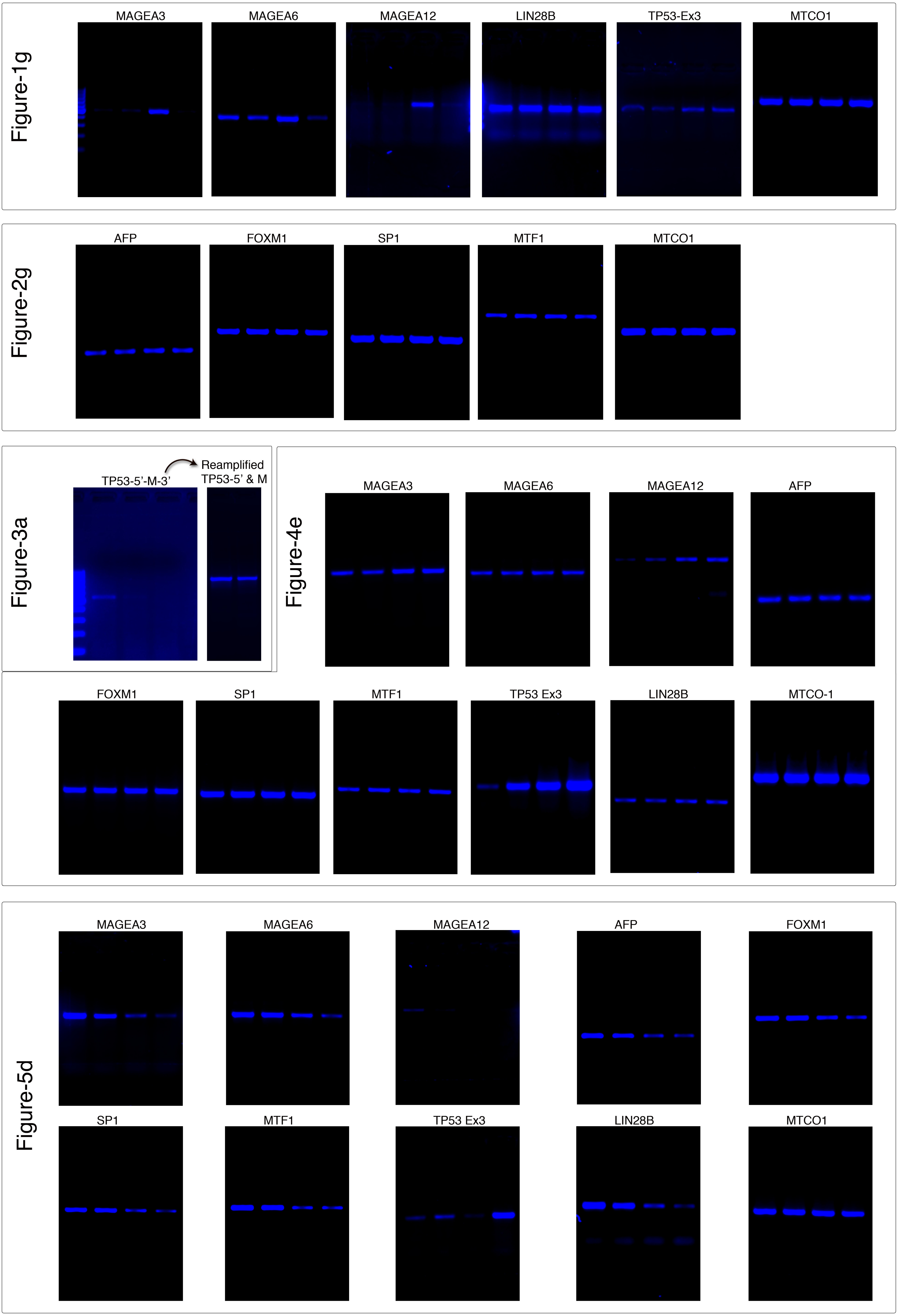

Supplementary figure-5

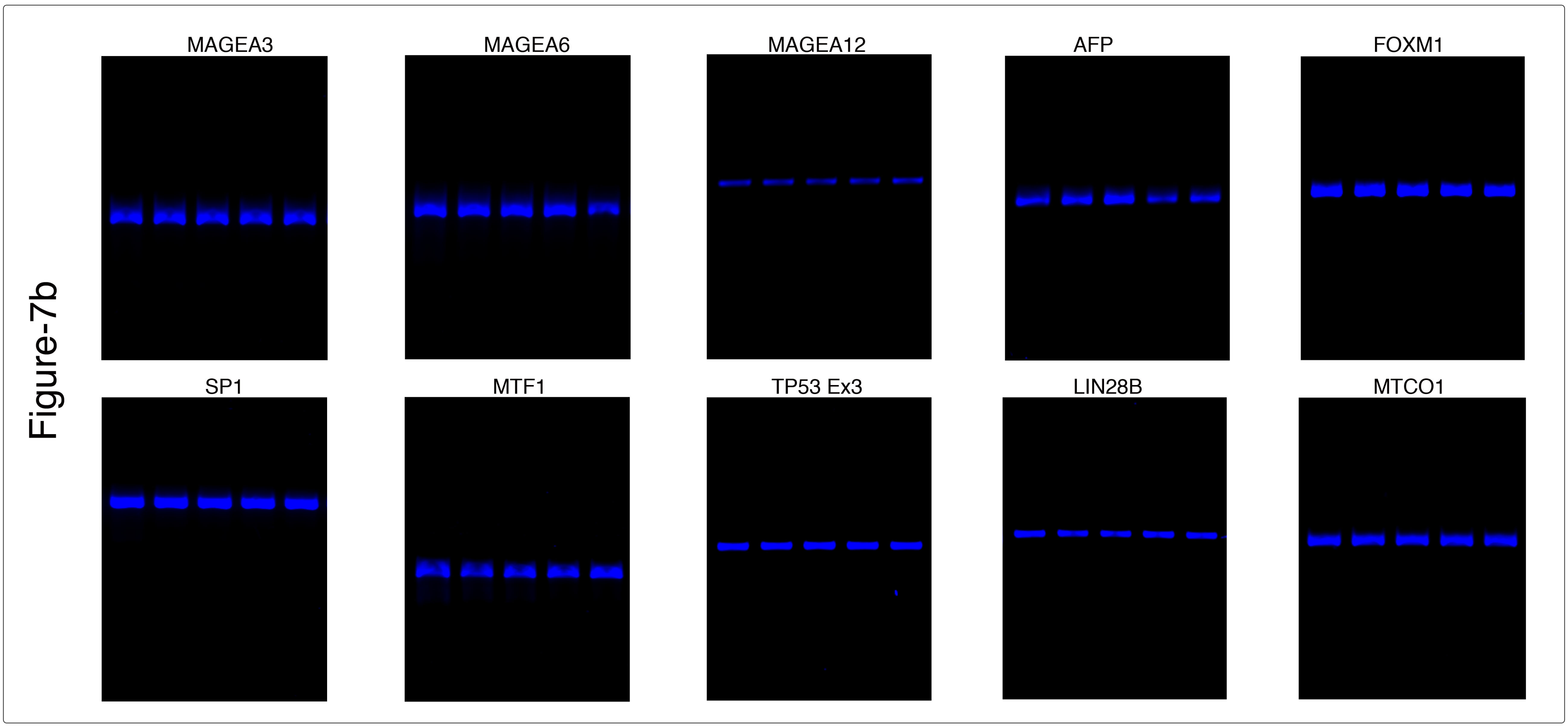

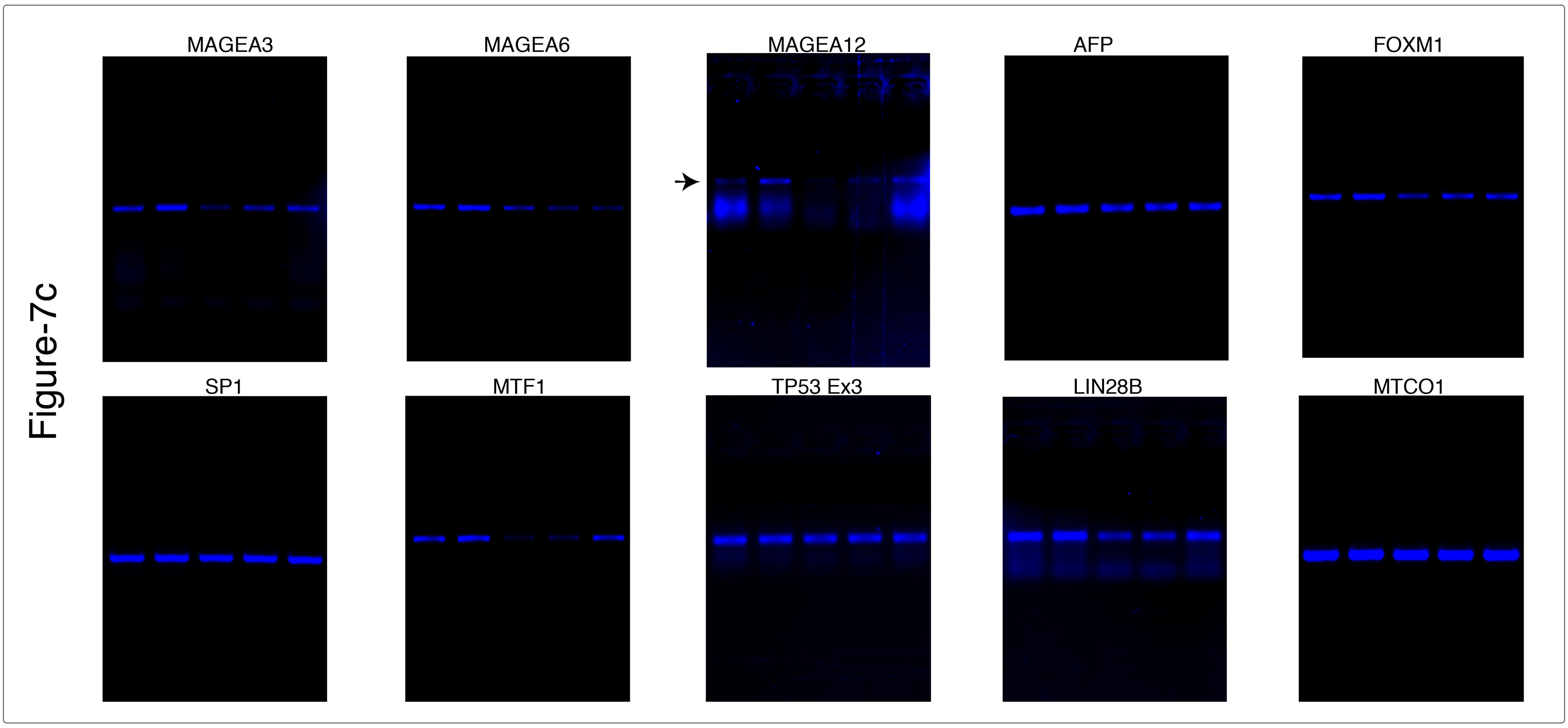

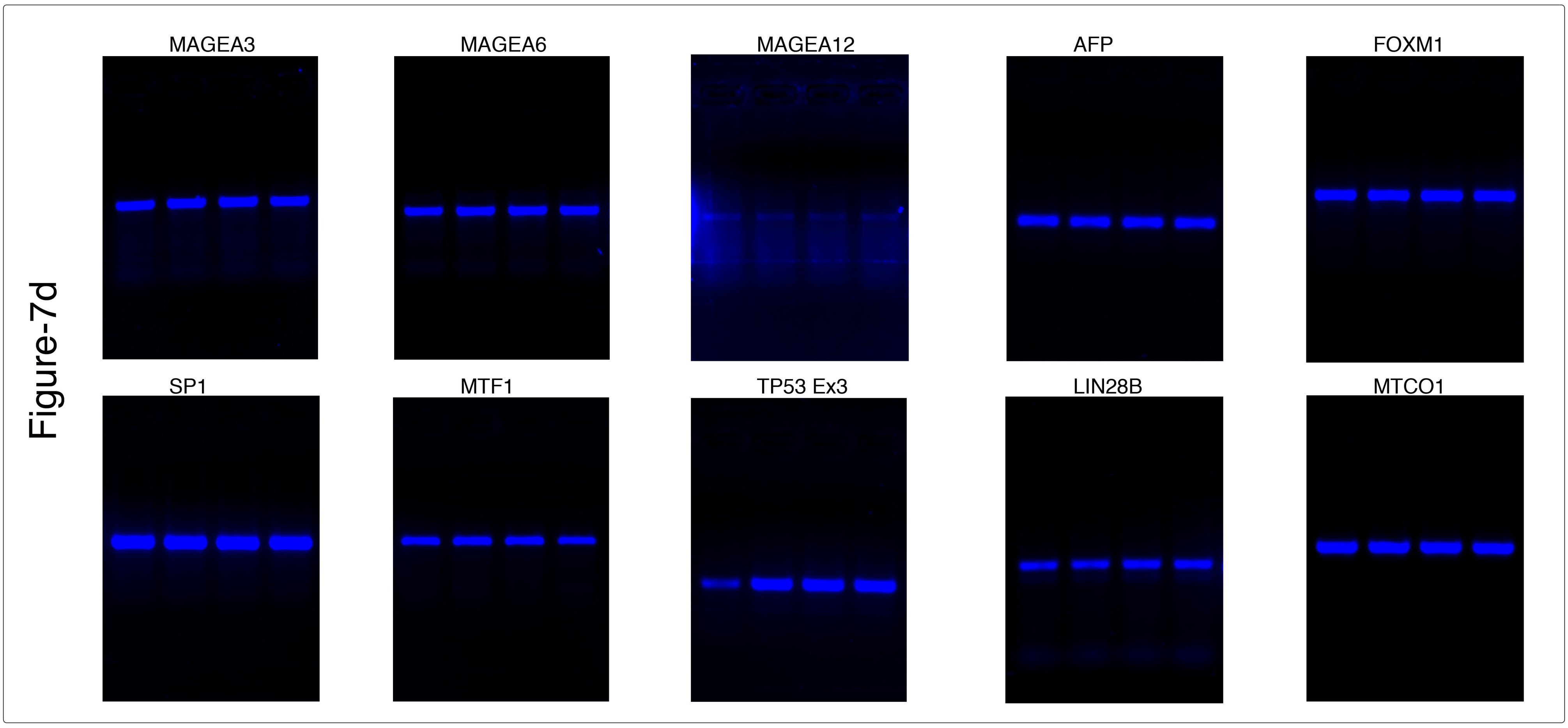

Legends for supplementary figures

Supplementary figure 1. MAGEA12 and CSAG1 share a common enhancer

Genome browser (hg19) image showing MAGEA12 and CSAG1 share a common enhancer highlighted by a red box and blue peaks of H3K27Ac. The gene direction is marked by arrows. Enhancers are being bidirectional usually it is common that two genes in opposite direction are often regulated by a common enhancer in human genome.

Supplementary figure 2. MAGEA-3, 6 and 12 are expressed more in p53 transcription defective cell lines

a-b, CCLE miRNA-seq data of 28 cell lines that display similar levels of C19MC expression (A) but show contrasting p53TCTI signature (B). **c**, CCLE RNA-seq expression analysis of MAGEA-3, MAGEA-6 and MAGEA-12 in 14 p53TC and 14 p53TI cell lines.

Supplementary figure 3. Dose finding for Zinc chloride in Hep3B cells

Hep3B parental cells were treated with various doses of $ZnCl_2$ for 24 hrs and observed for the effects on morphology. The pyknotic and non-pyknotic cells were counted to calculate the percentage of pyknosis.

Supplementary figure 4. Original RT-PCR gels of main figures. Part-1

Supplementary figure 5. Original RT-PCR gels of main figures Part-2

Supplementary table-1: p53TCTI dataset

p53TC TCGA-LIHC sample IDs	p53TI TCGA-LIHC sample IDs
TCGA-BC-A10S-01A-22R-A131-07	TCGA-BC-4072-01B-11R-A155-07
TCGA-BC-A10T-01A-11R-A131-07	TCGA-BC-4073-01B-02R-A131-07
TCGA-BC-A10X-01A-11R-A131-07	TCGA-BC-A10Y-01A-11R-A131-07
TCGA-BC-A110-01A-11R-A131-07	TCGA-BC-A216-01A-11R-A155-07
TCGA-BC-A69I-01A-11R-A311-07	TCGA-BC-A217-01A-11R-A155-07
TCGA-BW-A5NO-01A-11R-A27V-07	TCGA-BC-A3KG-01A-11R-A213-07
TCGA-CC-A7IH-01A-11R-A33J-07	TCGA-BW-A5NQ-01A-11R-A27V-07
TCGA-CC-A7IL-01A-11R-A33R-07	TCGA-CC-5258-01A-01R-A131-07
TCGA-DD-A116-01A-11R-A131-07	TCGA-CC-5263-01A-01R-A131-07
TCGA-DD-A11B-01A-11R-A131-07	TCGA-CC-5264-01A-01R-A131-07
TCGA-DD-A11D-01A-11R-A131-07	TCGA-CC-A1HT-01A-11R-A131-07
TCGA-DD-A1EB-01A-11R-A131-07	TCGA-CC-A3M9-01A-11R-A213-07
TCGA-DD-A1ED-01A-11R-A155-07	TCGA-CC-A3MA-01A-11R-A213-07
TCGA-DD-A39W-01A-11R-A213-07	TCGA-CC-A3MB-01A-11R-A213-07
TCGA-DD-A3A2-01A-11R-A213-07	TCGA-CC-A5UD-01A-11R-A28V-07
TCGA-DD-A3A4-01A-11R-A22L-07	TCGA-CC-A7IF-01A-11R-A33J-07
TCGA-DD-A3A8-01A-11R-A22L-07	TCGA-CC-A7IG-01A-11R-A33J-07
TCGA-DD-A4NB-01A-12R-A266-07	TCGA-CC-A7II-01A-11R-A33J-07
TCGA-DD-A4NI-01A-11R-A27V-07	TCGA-CC-A7IJ-01A-11R-A33R-07
TCGA-DD-A4NL-01A-11R-A28V-07	TCGA-CC-A7IK-01A-12R-A33R-07
TCGA-DD-A4NP-01A-11R-A28V-07	TCGA-DD-A114-01A-11R-A131-07
TCGA-DD-A4NS-01A-11R-A311-07	TCGA-DD-A1EG-01A-11R-A213-07
TCGA-DD-A4NV-01A-11R-A311-07	TCGA-DD-A1EI-01A-11R-A131-07
TCGA-DD-A73A-01A-12R-A32O-07	TCGA-DD-A4NN-01A-11R-A28V-07
TCGA-DD-A73C-01A-12R-A33J-07	TCGA-DD-A4NQ-01A-21R-A28V-07
TCGA-DD-A73D-01A-12R-A32O-07	TCGA-ED-A7PZ-01A-11R-A33R-07
TCGA-DD-A73E-01A-12R-A32O-07	TCGA-EP-A2KA-01A-11R-A180-07
TCGA-ED-A4XI-01A-11R-A266-07	TCGA-EP-A2KB-01A-11R-A180-07
TCGA-ED-A627-01A-12R-A311-07	TCGA-FV-A3I1-01A-11R-A22L-07
TCGA-EP-A12J-01A-11R-A131-07	TCGA-FV-A4ZQ-01A-11R-A266-07
TCGA-EP-A26S-01A-11R-A16W-07	TCGA-G3-A25X-01A-11R-A16W-07
TCGA-EP-A2KC-01A-11R-A213-07	TCGA-G3-A5SI-01A-31R-A27V-07
TCGA-ES-A2HS-01A-11R-A180-07	TCGA-G3-A7M6-01A-11R-A33R-07
TCGA-G3-A25V-01A-11R-A16W-07	TCGA-G3-A7M9-01A-23R-A352-07
TCGA-G3-A3CH-01A-11R-A22L-07	TCGA-K7-A5RG-01A-11R-A28V-07
TCGA-G3-A3CI-01A-11R-A213-07	TCGA-MI-A75I-01A-11R-A32O-07
TCGA-G3-A3CK-01A-11R-A213-07	TCGA-QA-A7B7-01A-11R-A32O-07
TCGA-G3-A5SL-01A-11R-A27V-07	TCGA-RC-A6M6-01A-11R-A32O-07
TCGA-G3-A6UC-01A-21R-A33J-07	TCGA-RG-A7D4-01A-12R-A33R-07
TCGA-G3-A7M5-01A-11R-A33R-07	TCGA-UB-A7MB-01A-11R-A33R-07
TCGA-G3-A7M7-01A-12R-A352-07	TCGA-UB-A7MC-01A-11R-A33R-07
TCGA-G3-A7M8-01A-11R-A33R-07	TCGA-UB-A7MF-01A-11R-A33J-07
TCGA-K7-A5RF-01A-11R-A28V-07	
TCGA-K7-A6G5-01A-11R-A311-07	
TCGA-KR-A7K0-01A-12R-A33R-07	
TCGA-MI-A75E-01A-11R-A32O-07	
TCGA-MI-A75H-01A-11R-A32O-07	
TCGA-MR-A520-01A-11R-A266-07	
TCGA-NI-A4U2-01A-11R-A28V-07	
TCGA-RC-A6M4-01A-11R-A32O-07	
TCGA-RC-A6M5-01A-11R-A32O-07	

Supplementary Table-2	RT-PCR				
gene target	Forward primer sequence	Reverse primer sequence	Product size	Annealing temperature used	Betaine used for PCR [Final conc.]
MAGEA3	5'-CTTGAGCAGAGGAGTCAGCACTGC-3'	5'-AGACCAGCTGCAAGGAACTGGAAG-3'	478 bp	60°C	1.5M for cDNA and 1M for RT-PCR
MAGEA6	5'-TGCCTCTTGAGCAGAGGAGTCAGC-3'	5'-AGCTCGATGCCAAAGACCAGCTGC-3'	496 bp	60°C	1.5M for cDNA and 1M for RT-PCR
MAGEA12	5'-AGGAGTCAGCACTGCAAGCCTGAG-3'	5'-TCCTGGAAATTTCTGATGACACTCC-3'	419 bp	60°C	1.5M for cDNA and 1M for RT-PCR
LIN28B	5'-TGGTGGCCTTGATCATCATGCTAAG-3'	5'-TTGCTTTGCTCTTCTGGTGCTATAG-3'	321 bp	60°C	1.5M for cDNA and 1M for RT-PCR
TP53-Exon-3	5'-ATGATTTGATGCTGTCCCCGGACG-3'	5'-CGTGCAAGTCACAGACTTGGCTG-3'	250 bp	60°C	1.5M for cDNA and 1M for RT-PCR
MTCO1	5'-ATGAGCTGGAGTCCTAGGCACAGC-3'	5'-AACCTGTTCCTGCTCCGGCCTCC-3'	305 bp	60°C	1.5M for cDNA and 1M for RT-PCR
AFP	5'-CCTGCCTTTCTGGAAGAACTTTGCC-3'	5'-TGTTCATGAATGTCTCCCTGTCTTC-3'	208 bp	60°C	1.5M for cDNA and 1M for RT-PCR
FOXM1	5'-CTTCTGCAGGACCAGGGAAAGAGG-3'	5'-TCAGAGGAGTCTGCTGGGAACGGG-3'	448 bp	60°C	1.5M for cDNA and 1M for RT-PCR
SP1	5'-ACTCCAGCAGGCTGTCCCCCTCC-3'	5'-AGACCCCTGTAGCCCACTGACCC-3'	403 bp	60°C	1.5M for cDNA and 1M for RT-PCR
MTF1	5'-TGCAGAGTCAGTCAGTGATGTTCCG-3'	5'-AGGATAGCTGTGTTGGGAGTTGGG-3'	483 bp	60°C	1.5M for cDNA and 1M for RT-PCR

	**TP53-FXR2 fusion gDNA PCR and sequencing**				
gene target	Forward TP53 intron primer sequence	Reverse FXR2 intron primer sequence	Product size	Annealing temperature used	Betaine used for PCR [Final conc.]
TP53-intron-FXR2 intron fusion	5'-TCAGGAGCCACTTGCCACCCTGC-3'	5'-TGCTCACTGCAACTTCTGCCTCCC-3'	425 bp for Hep3B cells	60°C	1M

	**TP53 mRNA RT-PCR and sequencing**				
gene target	Forward primer sequence	Reverse primer sequence	Product size	Annealing temperature used	Betaine used [Final conc.]
TP53-5' [5' end of RNA]	5'-GATGGGATTGGGGTTTTCCCCTCC-3'	5'-TAGCTGCCCTGGTAGGTTTTCTGG-3'	522 bp	60°C	1.5M for cDNA and 1M for RT-PCR
TP53-M [Middle piece of RNA]	5'-TTCCCAGAAAACCTACCAGGGCAG-3'	5'-TGTGCGCCGGTCTCTCCCAGGAC-3'	559 bp	60°C	1.5M for cDNA and 1M for RT-PCR
TP53-3' [3' end of RNA]	5'-CATCATCACACTGGAAGACTCCAGT-3'	5'-CAACTTGTTCAGTGGAGCCCCGGG-3'	586 bp	60°C	1.5M for cDNA and 1M for RT-PCR

Supplementary table-3: C19MC High versus low group clinical characteristics			
**C19MC groups: Clinical Characteristics**	**C19MC low**	**C19MC high**	**p-value**
**Age at Diagnosis:**			0.153
Mean	59	61	
Standard Deviation	12.77	11.85	
**HIGH AFP Levels (>300) at Diagnosis by percentage:**	13%	23%	2.15E-04
**Weight at diagnosis:**			0.673
Mean	73.11	75.87	
Standard Deviation	16.91	19.31	
**Race by percent:**			0.436
Asian	38%	35%	
African American/Black	5%	12%	
White	57%	53%	
**Sex by percent:**			0.701
Female	31%	36%	
Male	69%	64%	
**Risk Factors by patient case:**			0.445
Alcohol Use	16	22	
Hepatitis B	17	11	
Hepatitis C	8	10	
Pre -existing Medical Condition	4	3	
Tobacco Use	8	5	
Other	1	1	
**HepB/ HepC Incidence by percent:**	41%	35%	1*
**Pugh Score Category by patient case:**			0.0968
a	33	33	
b	4	0	
c	1	0	
**Ishak Score Category by patient case (Fibrosis):**			0.309
1, 2	5	4	
3, 4	2	5	
5	3	0	
6	9	11	
**Pathologic t category by patient case:**			0.457
t1	30	29	
t2	14	12	
t3	5	12	
t3a	7	4	
t3b	3	1	
t4	1	3	
**Patient Survivorship by percentage:**	79%	72%	1*

* No statistics from cBioportal, therefore statistics were calculated manually by integrating clinical and miRNASeq TCGA data then using unpaired Mann-Whitney Test on GraphPad.

Legends for supplementary tables

Supplementary table-1 - p53TCTI dataset IDs

TCGA-LIHC RNA-seq data were classified based on 30 signature p53 target genes and two large cluster that show clear differences in expression of p53 target genes were selected as p53TC and p53TI clusters. The supplementary table provides the sample IDs of this dataset.

Supplementary Table-2 List of primer sequences and PCR conditions

The primers used for the RT-PCR reactions, p53 mutation analysis, fusion amplification and sequencing were listed in supplementary table-2 with annealing temperatures and the usage of betaine for PCR reactions as most of the targets are highly GC rich in nature.

Supplementary Table-3 Clinical characteristics of C19MC high versus low groups of TCGA dataset

C19MC high versus low expressing patient TCGA barcode IDs were fed into cBioportal to get overall statistics of clinical characteristics between groups.
